# Clinical impact of panel-based error-corrected next generation sequencing versus flow cytometry to detect measurable residual disease (MRD) in acute myeloid leukemia (AML)

**DOI:** 10.1038/s41375-021-01131-6

**Published:** 2021-02-08

**Authors:** Nikhil Patkar, Chinmayee Kakirde, Anam Fatima Shaikh, Rakhi Salve, Prasanna Bhanshe, Gaurav Chatterjee, Sweta Rajpal, Swapnali Joshi, Shruti Chaudhary, Rohan Kodgule, Sitaram Ghoghale, Nilesh Deshpande, Dhanalaxmi Shetty, Syed Hasan Khizer, Hasmukh Jain, Bhausaheb Bagal, Hari Menon, Navin Khattry, Manju Sengar, Prashant Tembhare, Papagudi Subramanian, Sumeet Gujral

**Affiliations:** 1 Haematopathology Laboratory, ACTREC, Tata Memorial Centre, Navi Mumbai, India; 2 Homi Bhabha National Institute (HBNI), Mumbai, India; 3 Dept of Cytogenetics, ACTREC, Tata Memorial Centre, Navi Mumbai, India; 4 Adult Haematolymphoid Disease Management Group, Tata Memorial Centre, Mumbai, India; 5 Haemato-Oncology, CyteCare Cancer Hospital, Bangalore, India

**Keywords:** Translational research, Cancer genomics

## Abstract

We accrued 201 patients of adult AML treated with conventional therapy, in morphological remission, and evaluated MRD using sensitive error-corrected next generation sequencing (NGS-MRD) and multiparameter flow cytometry (FCM-MRD) at the end of induction (PI) and consolidation (PC). Nearly 71% of patients were PI NGS-MRD^+^ and 40.9% PC NGS-MRD^+^ (median VAF 0.76%). NGS-MRD^+^ patients had a significantly higher cumulative incidence of relapse (*p* = 0.003), inferior overall survival (*p* = 0.001) and relapse free survival (*p* < 0.001) as compared to NGS-MRD^−^ patients. NGS-MRD was predictive of inferior outcome in intermediate cytogenetic risk and demonstrated potential in favorable cytogenetic risk AML. PI NGS-MRD^−^ patients had a significantly improved survival as compared to patients who became NGS-MRD^−^ subsequently indicating that kinetics of NGS-MRD clearance was of paramount importance. NGS-MRD identified over 80% of cases identified by flow cytometry at PI time point whereas FCM identified 49.3% identified by NGS. Only a fraction of cases were NGS-MRD^−^ but FCM-MRD^+^. NGS-MRD provided additional information of the risk of relapse when compared to FCM-MRD. We demonstrate a widely applicable, scalable NGS-MRD approach that is clinically informative and synergistic to FCM-MRD in AML treated with conventional therapies. Maximum clinical utility may be leveraged by combining FCM and NGS-MRD modalities.

## Introduction

Acute myeloid leukemia is a disease characterized by heterogeneous biology resulting in varying clinical outcomes including relapse [1, 2]. There are limited novel treatment options such as targeted therapies using *FLT3* or *IDH2* inhibitors and most patients are treated based on the presumptive risk of relapse [3, 4]. This risk- adapted therapy typically considers standard cytogenetic and molecular variables as recommended by the European Leukemia Net [5]. Based on this risk, patients are recommended treatment with conventional (induction and consolidation) regimens or offered intensive therapy such as allogeneic bone marrow transplantation (aBMT) after achievement of remission. Amongst these, the largest group remains as intermediate risk AML characterized by non-uniform clinical outcomes.

The monitoring of a patient’s response to chemotherapy, called, measurable residual disease (MRD) is one of the most important predictors of outcome in hematological malignancies. Several investigators have demonstrated the clinical utility of flow cytometry based MRD detection (FCM-MRD) in AML at early chemotherapy time points as well as in a pre-transplant setting [6–14]. Although universally applicable, FCM-MRD suffers from suboptimal ability to predict relapse in AML compared to precursor B lineage acute lymphoblastic leukemia. A diverse array of sensitive molecular methods have been used to detect MRD in AML such as real time PCR [15] and droplet digital PCR [16]. These are useful for monitoring of individual gene mutations such as AML with mutated *NPM1* [17, 18] and chimeric gene fusions such as *RUNX1-RUNX1T1* [19]. Next generation sequencing (NGS) is a promising tool for sensitive MRD monitoring and has been used successfully to monitor *NPM1* [20, 21], *RUNX1,* [22] and *FLT3* [23] mutations as well as chimeric gene fusions [24, 25]. Amongst these, targets such as *NPM1* or chimeric gene fusions are highly stable between diagnosis and relapse and not particularly vulnerable to clonal evolution.

DNA-based focused target enrichment strategies (gene panels) are an attractive solution to detect MRD using NGS (NGS-MRD) as they can be applied to a broader population of patients as compared to single gene molecular testing [26–29].

However, short read sequencers are inherently prone to base calling errors limiting variant calling at 3–5% variant allele fraction (VAF) [30]. Although acceptable for diagnostic molecular pathology, this is undesirable assay performance for the detection of MRD. Error-corrected sequencing involves the physical incorporation of random oligonucleotides or unique molecular identifiers (UMI) at the library preparation stage prior to amplification of DNA. This allows us to tag individual DNA molecules with an unique molecular fingerprint [31, 32]. Such approaches have been used for myelodysplastic syndromes [33] and for pre-transplant MRD monitoring of AML as demonstrated by Thol and colleagues [34]. Thol utilized a sensitive patient-specific mutation tracking approach using UMI-based MRD detection. Although applicable to a broad spectrum of AML mutations, a tailored approach poses logistical and regulatory hurdles towards prospective MRD testing especially for early MRD time points.

In this study, we have evaluated the clinical utility of error-corrected NGS to detect MRD in AML using single molecule molecular inversion probes (smMIPS) [31, 35]. Each smMIP contains an 8 bp UMI and binds to a single molecule of DNA. Using consensus sequence-based variant calling we can detect somatic mutations including small indels in a sensitive manner. We demonstrate that error-corrected NGS-MRD at early time points in therapy is significantly predictive of outcome in patients of AML treated with conventional therapies. Furthermore, we systematically compare multicolor FCM-MRD with error-corrected NGS-MRD and assess the clinical utility of these two assays in a cohort of AML.

## Methods

### Patient characteristics, treatment and MRD sampling

The study was approved by the institutional ethics committee (IEC-III project 163) and participants were accrued after informed consent. A total of 393 adult patients of AML, diagnosed as per 2008 WHO criteria, were accrued in this study over a period of 6 years (Feb 2013 to May 2019). Cytogenetic (FISH and karyotyping) workup was performed as previously described [9, 21]. Somatic mutations at diagnosis were evaluated using a smMIPS based 50 gene myeloid panel as described previously [36]. We describe an NGS-MRD approach that was applicable to more than 80% of patients in this AML cohort [327 out of 393 AMLs, median two mutations per case (range 1–6 trackable mutations); Supplementary Fig. 1]. Of those (*n* = 319) achieving morphological CR, the smMIPS MRD panel was applicable to 266 (83.4%). MRD assessment could be performed in 201 adult patients of AML (enrollment flow chart in Supplementary Fig. 2). A summary of the clinical and laboratory characteristics of these 201 patients can be seen in Table 1.

**Table 1:** Summary of clinical, laboratory, and MRD characteristics of patients accrued in this study.

Parameter	Observation (%)
Demographics:	
Age	Range: 18–63 years Median: 36 years
Sex	Male: Female: 1.48: 1
Clinical characteristics:	
Total number of patients accrued	201
Remission characteristics:	
Complete remission (CR)	31
CR with incomplete hematologic recovery (CRi)	170
Bone marrow transplantation:	
Patients who underwent BMT	15
Laboratory characteristics:	
Blood counts at presentation	
1. More than 50,000/mm^3^	49
2. Less than 50,000/mm^3^	152
Classification according to cytogenetic risk:	
Favorable risk	48 (23.9%)
Intermediate risk	136 (67.7%)
Poor risk	17 (8.5%)
Post Induction flow MRD (*n* = 200):	
MRD positive	88 (44.0%)
MRD negative	112 (56.0%)
Post consolidation flow MRD (*n* = 98):	
MRD positive	21 (21.4%)
MRD negative	77 (78.6%)
Post induction NGS-MRD (*n* = 196):	
MRD positive	139 (70.9%)
MRD negative	57 (29.1%)
Post consolidation NGS-MRD (*n* = 127):	
MRD positive	52 (40.9%)
MRD negative	75 (59.1%)
Comparative analysis of FCM-MRD and NGS-MRD (post induction; *n* = 195)	
NGS-MRD^+^ FCM-MRD^+^	68 (34.9%)
NGS-MRD^+^ FCM-MRD^−^	70 (35.9%)
NGS-MRD^−^ FCM-MRD^+^	17 (8.7%)
NGS-MRD^−^ FCM-MRD^−^	40 (20.5%)
Comparative analysis of FCM-MRD and NGS-MRD (post consolidation; *n* = 87)	
NGS-MRD^+^ FCM-MRD^+^	09 (10.3%)
NGS-MRD^+^ FCM-MRD^-^	28 (32.2%)
NGS-MRD^−^ FCM-MRD^+^	08 (9.2%)
NGS-MRD^−^ FCM-MRD^−^	42 (48.3%)

All patients were treated with conventional “3 + 7” induction chemotherapy and further treated with high dose cytarabine (HiDAC) or allogeneic bone marrow transplantation (aBMT), if feasible [36]. Only 15 patients received aBMT and their outcome was not different from the rest with respect to OS and RFS (p = not significant; Supplementary Fig. 3) and are not considered separately. Sample for FCM-MRD was obtained from the bone marrow at the end of induction (PI; *n* = 200) and end of first consolidation cycle (PC, *n* = 98). NGS-MRD sample also obtained at the same time points (PI-196; PC-127) from the bone marrow (*n* = 269; PI:181 and PC:88) or peripheral blood (*n* = 51; PI:15 and PC:36).

### Detection of MRD using error-corrected NGS (NGS-MRD)

We created a 34-gene panel comprising of a pool of 302 smMIPS (as seen in Supplementary Table 1). In brief, this panel covers regions of 34 commonly mutated genes in AML (*ATM, BCOR, DNMT3A, EZH2, FLT3, GATA1, GATA2, IDH1, IDH2, JAK2, KDM6A, KIT, KMT2D, KRAS, NF1, NOTCH1, NOTCH2, NPM1, NRAS, PHF6, PTPN11, RAD21, RUNX1, SETBP1, SF3B1, SH2B3, SMC1A, SRSF2, STAG2, TET2, TP53, U2AF1, WT1, ZRSR2*). The panel was rebalanced (Supplementary Fig. 4) to ensure uniform capture across regions. Approximately 600 ng of genomic DNA was captured, treated with exonucleases, and PCR amplified to create a sequencing ready library. Details pertaining to smMIPS design, assay standardization, and sequencing are detailed in supplementary methods.

Reads were demultiplexed, trimmed, paired end assembled, and mapped to the human genome (build hg19). Singleton reads (originating from one UMI) were discarded, and consensus family-based variant calling performed using tools described in supplementary methods. We then created a site and mutation-specific error model to ascertain the relevance of an observed variant at each site [35]. Criteria for variant calling using the smMIPS MRD assay are described in supplementary methods. *FLT3*-ITD were detected using a novel one-step PCR-based NGS assay (see Supplementary Table 3). Variants were detected using a recently described algorithm for the accurate detection of *FLT3*-ITD [37]. *NPM1* mutations were additionally tracked using an ultrasensitive orthogonal *NPM1* MRD assay [21].

### Detection of MRD using multicolour flow cytometry (FCM-MRD)

FCM-MRD was detected using a previously described 10-color two-tube MRD assay [9, 21, 36, 38]. This approach uses a combination of leukemia associated immunophenotype and difference from normal approaches to detect MRD in AML.

### Endpoints and statistical analyses

Overall survival (OS) and relapse free survival (RFS) were calculated as previously described [9, 21, 36]. The prognostic impact of NGS and FCM-MRD assays on OS and RFS was computed using the Kaplan–Meier method and compared using log-rank test. Prognostic relevance of individual gene mutations seen at baseline in patients of AML were assessed and variables found to be significantly predictive of outcome were included in multivariate analysis. Multivariate analysis was performed using the cox proportional-hazards regression analysis that considered FCM-MRD and NGS-MRD. Separate models were constructed for post induction and post consolidation MRD time points. Grey test was used to compare the cumulative incidences of relapse (CIR) and non-relapse mortality (NRM) using “cmprsk” module in R [39]. The same module was used to generate representative graphs. Competing risk regression modeling was performed using cause-specific hazard approach to determine the different rates of relapse in the presence of covariates [40]. Positive predictive (PPV) and negative predictive value (NPV) were calculated as described in [34]. Accuracy was calculated using the formula: accuracy = sensitivity × prevalence + specificity × (1 − prevalence) [41].

## Results

The median follow-up of the cohort was 42.3 months. The median OS was 35.9 months (95% CI 27.2–42.8) and median RFS was 21.6 months (95% CI 17.0–28.9) months. Additional patient characteristics can be seen in Table 1.

### Next generation sequencing based AML MRD detection

**Fig. 1:**
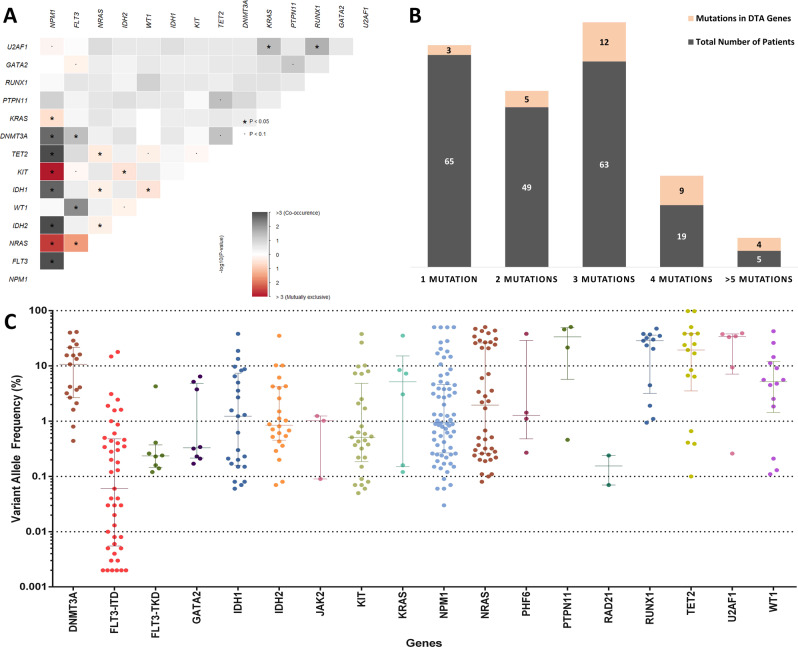
Somatic mutations in AML detected at diagnosis and during therapy. **A** The interaction of mutations at baseline is demonstrated here using Fisher’s exact test. Co-occurrence is indicated in gray color and mutual exclusivity is indicated in red. **B** The total number of mutations detected per patient and the number of such patients in the cohort is displayed. The total number of mutations in *DNMT3A-TET2-ASXL1* genes is indicated here as a fraction. **C** Variant allele frequencies of mutations detected at MRD time points for patients of AML in morphological remission. The bars indicate median values with interquartile ranges.

We describe an NGS-MRD approach that was applicable to more than 80% of patients in this AML cohort [83.4% (*n* = 266) patients in morphological CR]. A co-occurrence plot indicating interactions of mutations tracked by NGS-MRD prior to therapy, can be seen in Fig. 1A. The applicability of this MRD panel, when patients (*n* = 201) are classified by cytogenetic risk is seen in supplementary Table 2. In a limit of detection experiment (Supplementary Fig. 5), we demonstrated that we could detect leukemic clones till a lower limit of 0.05% (0.03% for *NPM1* mutation). Error modeling of normal patients indicated a higher prevalence of C > T and G > A changes consistent with oxidative DNA damage (Supplementary Fig. 6) [35]. *FLT3*-ITD could be detected at a limit of 0.002% VAF (Supplementary Fig. 7). For smMIPS-based MRD, sequencing was performed at a median coverage of 14,728x (11,363x consensus coverage) whereas, for *FLT3*-MRD assay, the median coverage was 1,396,366x. A total of 344 mutations could be detected in 323 MRD samples (Fig. 1B, C) with a median VAF of 0.95% [0.76% after exclusion of mutations in *DNMT3A, TET2, ASXL1* (DTA) genes]. A median of two mutations could be detected per patient (range 1–4) at the end of induction.

**Fig. 2:**
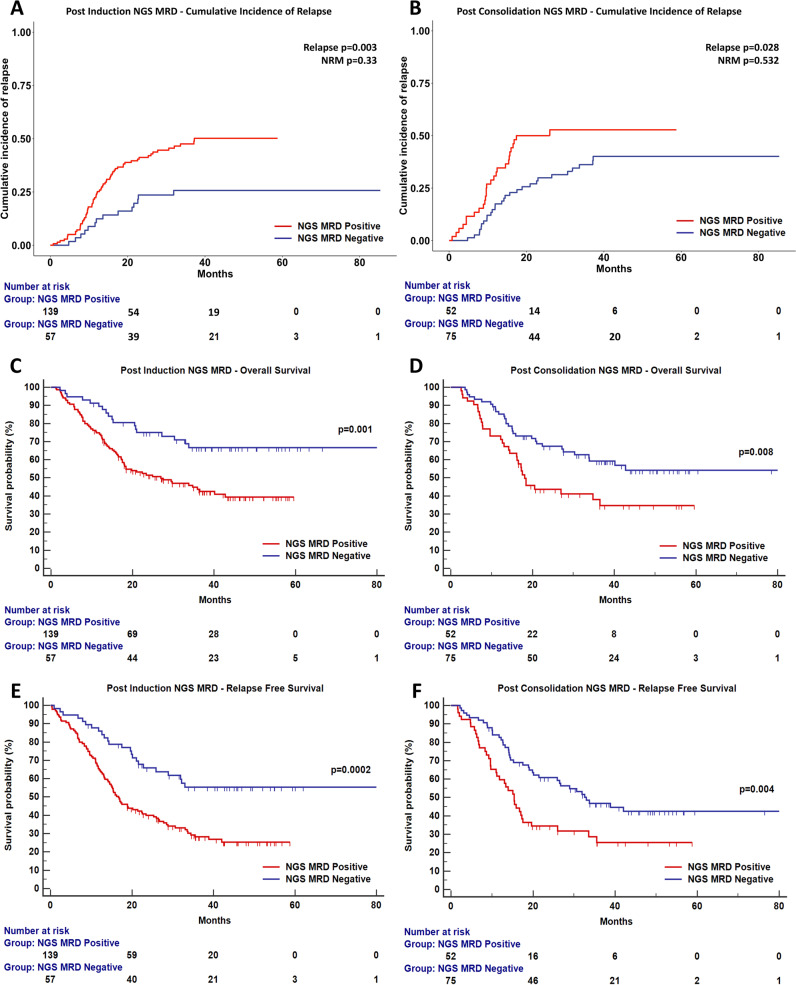
Clinical relevance of error-corrected NGS-MRD. Presence of NGS-MRD at post induction (**A**) and post consolidation time points (**B**) is associated with a higher cumulative incidence of relapse (CIR). Kaplan–Meyer plots indicate the clinical relevance of NGS-MRD with respect to OS and RFS at post induction (**C**, **E**) and post consolidation time points (**D**, **F**).

Nearly 71% (*n* = 139; 70.9%) of patients were NGS-MRD^+^ at the end of induction and 40.9% (*n* = 52) at the end of consolidation. NGS-MRD^+^ patients had a significantly higher CIR, OS, and RFS as compared to NGS-MRD^−^ patients at both MRD time points as seen in Fig. 2 and Tables 2, 3. The clinical impact of NGS-MRD when sample type was restricted to either the bone marrow (BM) or peripheral blood (PB) can be seen in Supplementary Figs. 8 and 9. The presence NGS-MRD was highly predictive of inferior OS and RFS for both MRD time points when patient samples were sourced from the BM. A similar trend can be postulated in PB sourced samples at PC time point but a definitive conclusion cannot be drawn due to limited numbers. NGS-MRD^+^ patients demonstrated an inferior outcome in intermediate cytogenetic risk (and a tendency in favorable risk) as seen in Supplementary Fig. 10.

**Table 2:** Prognostic influence of NGS-MRD, FCM-MRD and a combination of these modalities on the cumulative incidence of relapse (CIR).

Cumulative incidence of relapse						
Post induction NGS-MRD	Cumulative incidence of relapse (CIR)			Non-relapse mortality (NRM)		
	HR	3-year CIR (95% CI)	*P* value	HR	3-year cumulative incidence (95% CI)	*P* value
MRD negative	1	25.7% (14.9–37.9)	0.0005	1	18.7% (9.5–30.3)	0.1676
MRD positive	2.67	47.5% (38.7–55.8)		1.63	23.4% (16.6–30.8)	
Post consolidation NGS-MRD	Cumulative incidence of relapse			Non-relapse mortality		
	HR	3-year CIR (95% CI)	*P* value	HR	3-year cumulative incidence (95% CI)	*P* value
MRD negative	1	36.2% (25.1–47.4)	0.007	1	16.6% (9.0–26.1)	0.2071
MRD positive	2.03	52.8% (37.8–65.7)		1.57	19.2% (9.8–31.0)	
Post induction FCM-MRD	Cumulative incidence of relapse			Non-relapse mortality		
	HR	3-year CIR (95% CI)	*P* value	HR	3-year cumulative incidence (95% CI)	*P* value
MRD negative	1	33.6% (24.7–42.7)	0.0004	1	19.8% (12.7–28.0)	0.1260
MRD positive	2.17	49.8% (38.6–59.9)		1.63	25.2% (16.6–34.8)	
Post consolidation FCM-MRD	Cumulative incidence of relapse			Non-relapse mortality		
	HR	3-year CIR (95% CI)	*P* value	HR	3-year cumulative incidence (95% CI)	*P* value
MRD negative	1	35.9% (25.1–46.9)	0.0005	1	19.9% (11.7–29.7)	0.1618
MRD positive	3.09	61.9% (36.3–79.7)		1.86	23.8% (8.2–43.9)	
Comparative analysis of FCM-MRD and NGS-MRD (post induction)	Cumulative incidence of relapse			Non-relapse mortality		
	HR	3-year CIR (95% CI)	*P* value	HR	3-year cumulative incidence (95% CI)	*P* value
NGS-MRD^−^ FCM-MRD^−^	1	18.7% (8.0–32.7)	0.0001	1	13.8% (4.8–27.3)	0.2042
NGS-MRD^+^ FCM-MRD^−^	3.36	41.9% (29.6–53.7)		2.34	23.7% (14.2–34.6)	
NGS-MRD^−^ FCM-MRD^+^	4.04	41.2% (17.5–63.6)		3.07	29.4% (9.8–52.4)	
NGS-MRD^+^ FCM-MRD^+^	5.40	52.5% (39.6–63.9)		2.65	23.5% (14.2–34.2)	
Comparative analysis of FCM-MRD and NGS-MRD (post consolidation)	Cumulative incidence of relapse			Non-relapse mortality		
	HR	3-year CIR (95% CI)	*P* value	HR	3-year cumulative incidence (95% CI)	*P* value
NGS-MRD^−^ FCM-MRD^−^	1	24.8 % (12.6–39.0)	0.0001	1	22.0% (10.7–35.9)	0.1477
NGS-MRD^+^ FCM-MRD^−^	2.55	50.0% (29.9–67.1)		1.07	17.8% (6.3–34.1)	
NGS-MRD^−^ FCM-MRD^+^	5.39	87.5% (17.2–98.9)		-	0.0% (-)	
NGS-MRD^+^ FCM-MRD^+^	5.80	55.5% (14.7–83.5)		3.26	33.3% (5.7–65.6)	
Dual time point NGS-MRD	Cumulative incidence of relapse			Non-relapse mortality		
	HR	3-year CIR (95% CI)	*P* value	HR	3-year cumulative incidence (95% CI)	*P* value
MRD negative	1	27.8% (13.5–44.0)	0.01	1	15.9% (5.6–31.1)	0.6803
Either MRD positive	2.14	44.7% (28.6–59.7)		1.35	19.0% (8.8–32.2)	
MRD positive	2.90	52.9% (37.0–66.6)		1.63	17.4% (8.0–29.7)

**Table 3:** Difference in overall survival and relapse free survival between FCM-MRD, NGS-MRD.

Univariate cox analysis				
Post induction FCM-MRD	Overall survival (OS)		Relapse free survival (RFS)	
	HR (95% CI)	*P*	HR (95% CI)	*P*
MRD negative	1	0.0002	1	0.0008
MRD positive	2.1 (1.40–3.13)		1.8 (1.26–2.60)	
Post induction FCM-MRD	Overall survival (OS)		Relapse free survival (RFS)	
	HR (95% CI)	*P*	HR (95% CI)	*P*
MRD negative	Mean OS: 58.0 months; 95% CI (51.2–64.8 months), Median OS: Not reached	0.0002	Mean RFS: 47.0 months; 95% CI (40.2–53.8 months), Median RFS: 32.2 months 95% CI (22.4–42.0 months)	0.0008
MRD positive	Mean OS: 36.3 months; 95% CI (29.6–43.0 months), Median OS: 18.4 months; 95% CI (15.1–33.9 months)		Mean RFS: 28.4 months; 95% CI (22.6–34.3 months), Median RFS: 15.4 months; 95% CI (12.7–20.1 months)	
Post consolidation FCM-MRD	Overall survival (OS)		Relapse free survival (RFS)	
	HR (95% CI)	*P*	HR (95% CI)	*P*
MRD negative	1	0.04	1	0.001
MRD positive	1.9 (0.90–3.91)		2.4 (1.17–4.81)	
Post consolidation FCM-MRD	Overall survival (OS)		Relapse free survival (RFS)	
	HR (95% CI)	*P*	HR (95% CI)	*P*
MRD negative	Mean OS: 52.6 months; 95% CI (44.6–60.7 months), Median OS: Not reached	0.04	Mean RFS: 45.4 months; 95% CI (37.5–53.3 months), Median RFS: 31.9 months 95% CI (19.1–38.9 months)	0.001
MRD positive	Mean OS: 28.7 months; 95% CI (18.7–38.7 months), Median OS: 16.5 months; 95% CI (11.2–33.9 months)		Mean RFS: 19.4 months; 95% CI (11.6–27.2 months), Median RFS: 11.8 months; 95% CI (9.6–20.1 months)	
Post induction NGS-MRD	Overall survival (OS)		Relapse free survival (RFS)	
	HR (95% CI)	*P*	HR (95% CI)	*P*
MRD negative	1	0.001	1	0.0002
MRD positive	2.2 (1.47–3.42)		2.3 (1.58–3.31)	
Post induction NGS-MRD	Overall survival (OS)		Relapse free survival (RFS)	
	HR (95% CI)	*P*	HR (95% CI)	*P*
MRD negative	Mean OS: 63.2 months; 95% CI (54.4–72.0 months), Median OS: Not reached	0.001	Mean OS: 54.9 months; 95% CI (45.7–64.2 months), Median OS: Not reached	0.0002
MRD positive	Mean OS: 32.9 months; 95% CI (28.9–36.9 months), Median OS: 27.0 months; 95% CI (17.9–42.8 months)		Mean OS: 26.1 months; 95% CI (22.4–29.6 months), Median OS: 16.7 months; 95% CI (14.5–22.4 months)	
Post consolidation NGS-MRD	Overall survival (OS)		Relapse free survival (RFS)	
	HR (95% CI)	*P*	HR (95% CI)	*P*
MRD negative	1	0.008	1	0.004
MRD positive	1.9 (1.14–3.22)		1.9 (1.18–3.06)	
Post consolidation NGS-MRD	Overall survival (OS)		Relapse free survival (RFS)	
	HR (95% CI)	*P*	HR (95% CI)	*P*
MRD negative	Mean OS: 55.5 months; 95% CI (47.2–63.7 months), Median OS: Not reached	0.008	Mean OS: 46.9 months; 95% CI (38.8–54.9 months), Median OS: 33.0 months; 95% CI (21.4–42.0 months)	0.004
MRD positive	Mean OS: 30.1 months; 95% CI (23.7–36.5 months), Median OS: 18.1 months; 95% CI (16.1–36.5 months)		Mean OS: 24.6 months; 95% CI (18.7–30.6 months), Median OS: 15.4 months; 95% CI (11.2–17.5 months)	

*OS* overall survival, *RFS* relapse free survival, *CI* confidence interval.

Out of 122 patients in whom both (PI and PC) MRD time points were assessed, 83 patients were PI NGS-MRD^+^ and 46 were PC NGS-MRD^+^. A change in MRD profile occurred in 18 patients (39.13%). This included a loss of mutation in most cases (*n* = 14) and gain in the rest (Supplementary Fig. 11). There were five patients who were NGS-MRD^−^ at the end of induction but became NGS-MRD^+^ at end of consolidation. Of these, relapse was seen in two patients. It should be noted that this change in MRD mutation profile between two MRD time points is not conceptually the same as a genuine gain in mutation which was not present at diagnosis of AML. Patients who were NGS-MRD^−^ at all MRD time points had a significantly improved OS [HR 0.45; 95% CI 0.22–0.9; (*p* = 0.02)] and RFS [HR 0.49; 95% CI 0.27–0.89; (*p* = 0.01)] as compared to patients who became negative at the end of consolidation (Supplementary Fig. 12). Similarly, patients who were persistently NGS-MRD^+^ had a significantly inferior outcome as compared to patients who were MRD negative at both time points (Supplementary Fig. 13). There was no genetic difference observed between these two groups (Supplementary Fig. 14).

We could detect MRD in *NPM1* mutated AML using an orthogonal technique in 75 patients (23.2% of all samples; Supplementary Fig. 15). There was a good correlation observed with *NPM1* NGS-MRD assay (*R*^2^ = 0.71) at the limit of detection of the smMIPS MRD assay.

### FCM based AML MRD and comparison with NGS-MRD

**Fig. 3:**
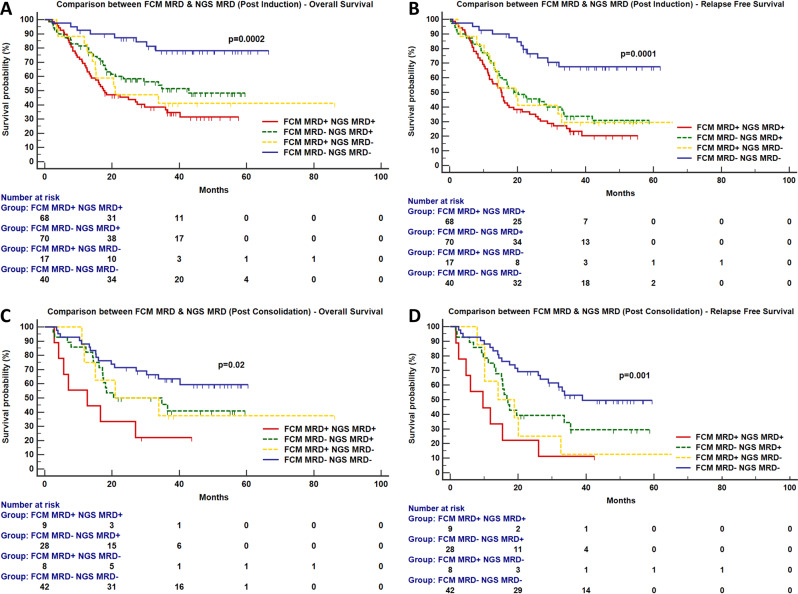
Comparison between FCM and NGS-MRD. The clinical relevance detection of MRD during complete remission when measured by FCM or error-corrected NGS at post induction (**A**, **B**) and post consolidation time points (**C**, **D**).

The presence of FCM-MRD was associated with inferior OS, RFS, and CIR at the end of induction and consolidation as detailed in Tables 2, 3 and Supplementary Fig. 16. On incorporating results combining both the MRD modalities, patients that were positive by both techniques (FCM^+^NGS^+^) had a significantly inferior outcome with respect to OS, RFS, and CIR at any MRD time point as compared to patients negative by both modalities as seen in Table 2 and supplementary Table 5 (Fig. 3). A comparison of the baseline mutational profiles between dual PI MRD positive (FCM^+^NGS^+^) and negative groups (FCM^−^NGS^−^) revealed a significantly higher (*p* = 0.04) prevalence of *RUNX1* mutations in the dual MRD positive subset (Supplementary Fig. 17). A total of 20 patients were (FCM^+^ NGS^−^) assessed at PI and/or PC time points. Their genetic profiles as well as MRD results (FCM/NGS) and eventual outcome are detailed in supplementary Table 6.

A total of 32 samples were sourced from PB for NGS-MRD (PI:15, PC:17) where FCM-MRD was measured in BM. Of these, 14 were FCM-MRD^-^ but were NGS-MRD^+^. Meanwhile, three patients were FCM-MRD^+^ but NGS-MRD^−^ as detailed in supplementary Table 7.

### Metrics for assay performance

The PPV and NPV metrics of end of induction NGS-MRD to predict relapse in AML were 70.5% and 57.89% respectively with an accuracy of 66.84%. FCM-MRD metrics at the end of induction were comparable for PPV (75%), NPV (48.2%), and accuracy to predict relapse (60%). At the PI time point, NGS-MRD identified 80% (68 out of 85) of cases classified as MRD positive by FCM, whereas FCM identified just 68 out of 138 cases (49.3%) identified by NGS. A detailed comparison of PPV, NPV, and accuracy of combinations of patients detected between these two assays can be seen in supplementary Table 8.

### Multivariate analysis

Multivariate analysis included high VAF (>11) *FLT3*-ITD, *RUNX1* mutation, poor risk cytogenetics along with FCM and NGS-MRD. The presence of high VAF *FLT3*-ITD, *RUNX1* mutation, FCM-MRD, and NGS-MRD were important in predicting outcome as seen in Table 4 for OS and RFS at the post induction MRD time point.

**Table 4:** Multivariate cox analysis for the presence of *FLT3*-ITD, *RUNX1* mutation, FCM-MRD and NGS-MRD at post induction and post consolidation time points for OS, RFS, and CIR.

Multivariate cox analysis				
	Overall survival (OS)		Relapse free survival (RFS)	
	HR (95% CI)	*P*	HR (95% CI)	*P*
*FLT3*-ITD mutation	2.41 (1.43–4.09)	0.001	2.30 (1.42–3.71)	0.0006
*RUNX1* mutation	2.76 (1.25–6.06)	0.012	2.14 (0.99–4.61)	0.05
Poor cytogenetic risk	1.46 (0.73–2.90)	0.276	1.42 (0.74–2.70)	0.284
PI FCM-MRD positive	1.88 (1.22–2.91)	0.004	1.70 (1.15–2.49)	0.007
PI NGS-MRD positive	1.99 (1.16–3.40)	0.012	2.01 (1.26–3.18)	0.003
	Cumulative incidence of relapse (CIR)			
	HR (95% CI)			*P*
*FLT3*-ITD mutation	1.64 (0.84–3.20)			0.14
*RUNX1* mutation	3.63 (1.31–10.0)			0.013
Poor cytogenetic risk	0.80 (0.34–1.86)			0.6
PI FCM-MRD positive	1.89 (1.17–3.03)			0.009
PI NGS-MRD positive	2.32 (1.28–4.20)			0.006
	Overall survival (OS)		Relapse free survival (RFS)	
	HR (95% CI)	*P*	HR (95% CI)	*P*
*FLT3*-ITD mutation	3.36 (1.56–7.23)	0.002	3.60 (1.75–7.37)	0.0005
*RUNX1* mutation	2.28 (0.62–8.42)	0.215	1.40 (0.39–4.94)	0.599
Poor cytogenetic risk	0.65 (0.23–1.81)	0.416	0.86 (0.35–2.10)	0.745
PC FCM-MRD positive	1.92 (0.95–3.87)	0.07	2.63 (1.41–4.90)	0.002
PC NGS-MRD positive	1.61 (0.86–3.03)	0.136	1.65 (0.94–2.89)	0.08
	Cumulative incidence of relapse (CIR)			
	HR (95% CI)			*P*
*FLT3*-ITD mutation	2.85 (1.07–7.61)			0.04
*RUNX1* mutation	5.09 (0.94–27.5)			0.06
Poor cytogenetic risk	1.24 (0.40–3.83)			0.7
PC FCM-MRD positive	3.90 (1.90–8.03)			<0.001
PC NGS-MRD positive	1.71 (0.86–3.38)			0.12

*OS* overall survival, *RFS* relapse free survival, *CIR* cumulative incidence of relapse, *PI* post induction, *PC* post induction.

## Discussion

Recently, Hourigan and colleagues [42] performed ultradeep sequencing using a 13-gene panel to detect MRD in AML. In a pioneering effort, they demonstrated an advantage of myeloablative conditioning in preventing relapse in an AML cohort based on NGS-MRD results. The authors, however, were unable to compare their results with other MRD assessment techniques. Here, we have assessed MRD at serial time points and have compared our results with 10-color FCM-MRD, which is a widely used technique for the assessment of response to chemotherapy [43]. We find that NGS-MRD is comparable in applicability and adds value, especially when a clear distinction of regenerating myeloid progenitors from leukemic blasts is absent.

In our manuscript we demonstrate that NGS- MRD identified over 80% of cases identified by flow cytometry at PI time point. On evaluating 17 discrepant cases (PI time point; 15 BM samples) which were NGS-MRD^−^ but FCM-MRD^+^, we observed that majority patients (10 out of 17) are alive or have died due to causes other than relapse indicating that these could have been false positives. Analysis of relapsed patients (*n* = 7) revealed that three samples had lower coverage (mean consensus coverage-8363x). In these cases, MRD detection could have been inaccurate due to suboptimal sensitivity. Two out of the remaining three discrepant cases were NGS-MRD^−^ at the end of consolidation but were NGS-MRD^+^ at the end of induction. A discrepant NGS-MRD result is unlikely to have therapeutic implications in these two patients.

We demonstrate that patients who are NGS-MRD^−^ at the end of induction are likely to have a superior outcome as compared to patients who subsequently become NGS-MRD^−^ (Supplementary Fig. 12). We have previously demonstrated that *NPM1* NGS-MRD values are comparable when simultaneously measured from the blood and bone marrow [21]. We observed a good correlation with a minimal loss of sensitivity when MRD measurements were made in the blood (median of 0.4 log difference of VAF levels when compared to BM) [21]. In this manuscript, we demonstrate that a blood sample may be acceptable for NGS-MRD when BM sampling is unfeasible.

Previously Jongen–Lavrencic and colleagues have demonstrated clinical utility of NGS to detect MRD in AML by using computational error correction to mitigate sequencing errors [27]. Such an approach, although easy to implement, discounts for batch effects and variability that occurs because of library clustering and batch-dependent PCR artefacts [32]. In that context, to the best of our knowledge, this is the first study to determine the clinical importance of (error-corrected, panel-based) NGS-MRD in AML treated with conventional therapies. Although our NGS-MRD strategy works in a majority of AML, we were curious about the genetic basis of cases (*n* = 65 out of 393) in which this strategy did not work. The cytogenetic and mutational landscape can be seen in Supplementary Fig. 18 and their outcome is detailed in supplementary methods. Many of these patients demonstrated favorable cytogenetic risk (49.2%) and nearly half did not show presence of any mutation at diagnosis (*n* = 29; 44.6%). Insight into rest of the cases revealed *ASXL2* as a recurrently mutated gene (*n* = 8,12.3%). Incorporation of *ASXL2* in future iterations of our panel will help in increasing the breadth of our approach. Alternative MRD monitoring approaches such as qPCR or UMI-based RNA sequencing [24, 25] to monitor chimeric gene fusions should be considered in favorable risk AML. This would be expected to increase the applicability of MRD detection to >90% of all AML patients in our cohort.

Consistent with previous reports, we find that in some patients, mutations in DTA genes are present at high VAF at MRD time points (Fig. 1) indicating an origin from an ancestral clone possibly originating from clonal haematopoesis [27, 34, 42]. In our series, a total of 33 patients had DTA mutations which were trackable by MRD panel (including three with a sole DTA mutation). Of these, 21 patients were NGS-MRD^+^ with persistent DTA mutations. The decision to label a patient as NGS-MRD^+^ in 19 patients was not based on the presence of DTA mutation but was made on other persistent non-DTA mutations. In two patients as no other mutation was trackable, the decision to label the patient as NGS-MRD^+^ was made based on a persistent *TET2* clone. The clinical relevance of NGS-MRD after exclusion of DTA mutations can be seen in Supplementary Fig. 19. No patient was considered as NGS-MRD^+^ based on persistence of DTA mutation at the end of consolidation.

Unlike amplicon-based [28, 34] approaches, the advantage of a smMIPS-based capture includes a stable panel which can be used across a spectrum of cases and no susceptibility to allelic skew or PCR-induced errors prior to incorporation of the UMI barcode. Disadvantages of smMIPS include poor performance for GC rich genes such as *CEBPA* gene and inability to capture low yield or poor quality of DNA (a problem not infrequently seen with MRD samples). The library preparation process is relatively low cost in nature and the overall process has a realistic turnaround time of 5–7 days. Our observation is that sensitivity in the clinic for most mutations is close to 0.1% VAF. A higher sensitivity can be obtained for complex indels such as *NPM1* and *FLT3*-ITD. This level of sensitivity (0.03% VAF) for *NPM1* mutated AML is at least one log lower than what is possible by RNA-based qPCR assays [17]. However, advantages over qPCR include the ability to monitor any *NPM1* mutation subtype in a single assay with uniform assay performance characteristics across all *NPM1* subtypes [44].

Improvements with sensitivity may be possible through duplex-sequencing based methods albeit at a much higher cost of sequencing [30]. Based on this data we find that mutations in *NPM1, FLT3, NRAS, KIT, IDH1, IDH2, WT1, RUNX1, GATA2, U2AF1*, and *PHF6* were most helpful in considering a patient as NGS-MRD^+^ (Supplementary Fig. 20). In our study, a vast majority (75.7%) of mutations monitored in patients with favorable cytogenetic risk include signaling pathway mutations (in *FLT3, KIT, NRAS* and *KRAS* genes) which could be susceptible to clonal evolution with targeted therapy. A limitation of our study is lack of orthogonal comparison with standard qPCR MRD tests for detection of fusion transcripts such as *RUNX1-RUNX1T1* or *CBFB-MYH11*.

To conclude, we demonstrate that panel-based error-corrected NGS-MRD is clinically relevant and synergistic in application to FCM-based AML MRD monitoring.

## Supplementary information

Supplementary Methods

Patient Dataset
