# Supplementary material for: Clinical impact of panel-based error-corrected next generation sequencing versus flow cytometry to detect measurable residual disease (MRD) in acute myeloid leukemia (AML)
Source: Leukemia. 2021 Feb 8;35(5):1392–404. doi: 10.1038/s41375-021-01131-6 (PMC8102181; doi:10.1038/s41375-021-01131-6)
Supplement: Supplementary file 1 — Supplementary Methods [file 41375_2021_1131_MOESM1_ESM.pdf]

Supplementary Methods

Patkar et al. Molecular MRD Detection in AML using Error Corrected NGS

1. Detection of AML MRD using smMIPS

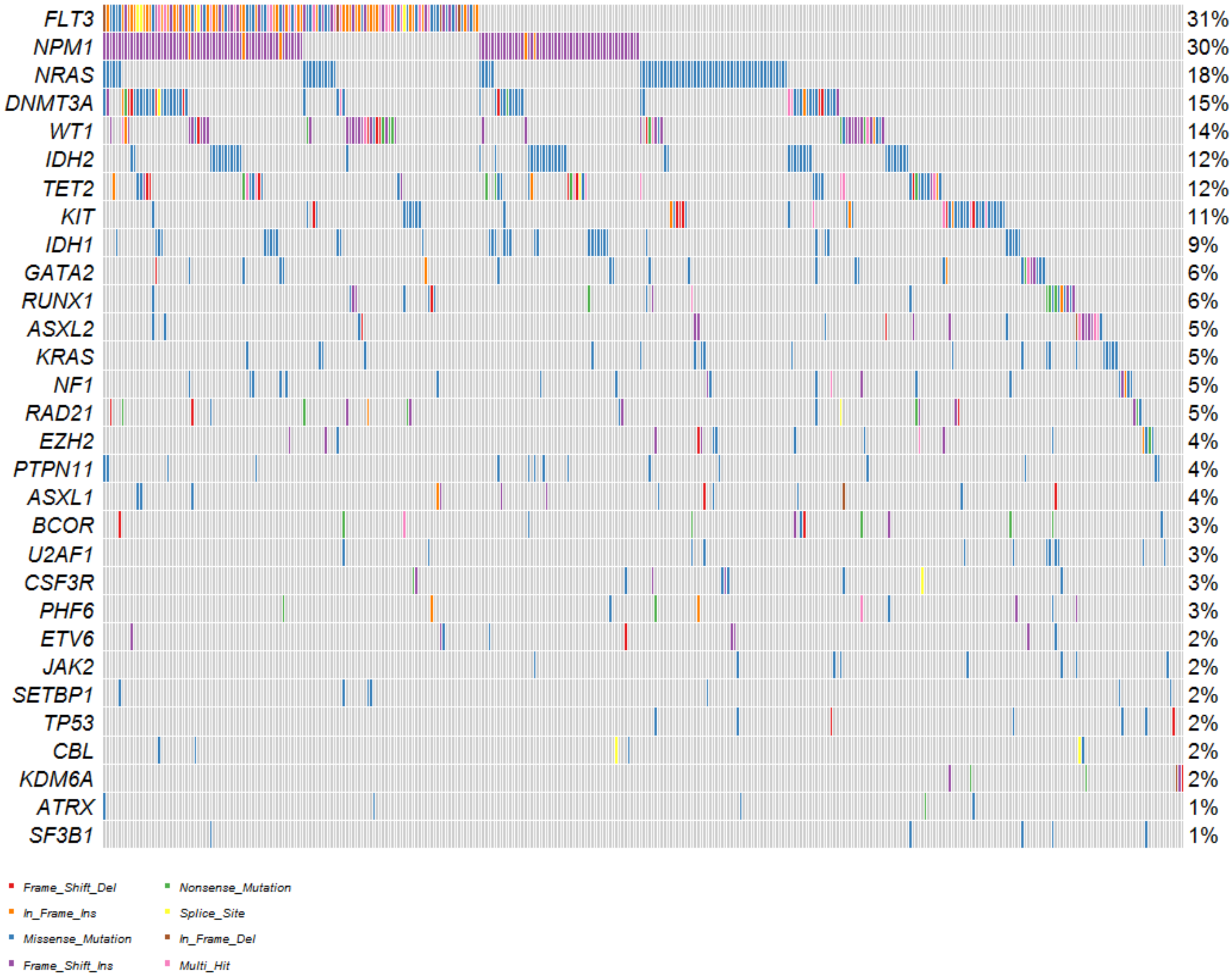

Supplementary Figure 1: Above oncoplot (on 393 patients) demonstrates the frequency of somatic mutations at diagnosis in AML detected using a 50 gene smMIPS myeloid panel. This genomic landscape formed the basis for designing the NGS MRD panel.

a. *Design of smMIPS*:

We reviewed somatic mutations in 393 patients of adult AML (Supplementary Figure 1) diagnosed at the Tata Memorial Centre as well as mutations in TCGA AML cohort (www.cbioportal.org). Based on this data we designed a 35 gene myeloid hotspot panel comprising of 302 single molecule molecular inversion probes (smMIPS). The genes covered by this "hot-spot" panel can be seen in Supplementary Table 1. The panel comprised of a main panel and an add on module which covered uncommon mutations. The latter panel was added if mutations (covered by addon panel) were present at diagnosis. smMIPS were designed using MIPgen[1] software with the parameters -max_capture_size 162, -min_capture_size 152, -logistic_priority_score 0.5. Extenstion arm length parameters were set between 18-21 and ligation arm length between 21-24. Each smMIP was designed to include a four basepair (bp) unique molecular identifier (UMI) at each end (total of 8bp degenerate nucleotide sequence per smMIP).

Supplementary Methods

Patkar et al. Molecular MRD Detection in AML using Error Corrected NGS

Recruitment of Patients in NGS-MRD Study

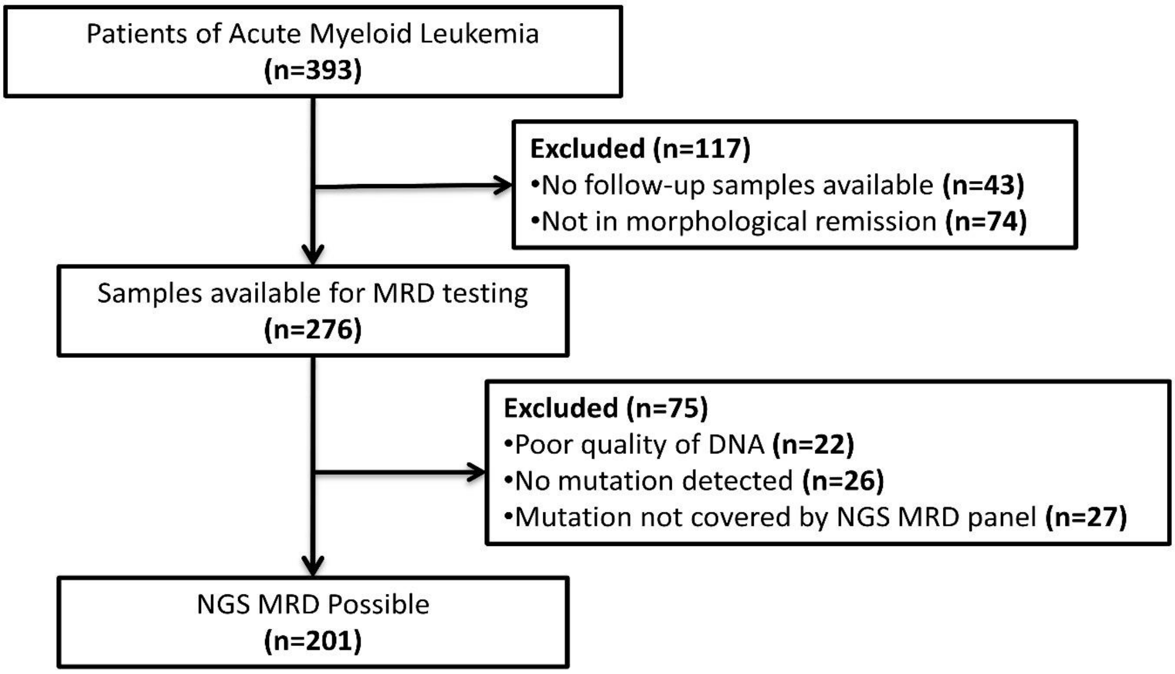

Supplementary Figure 2: Flow chart indicates recruitment of patients in AML NGS-MRD study.

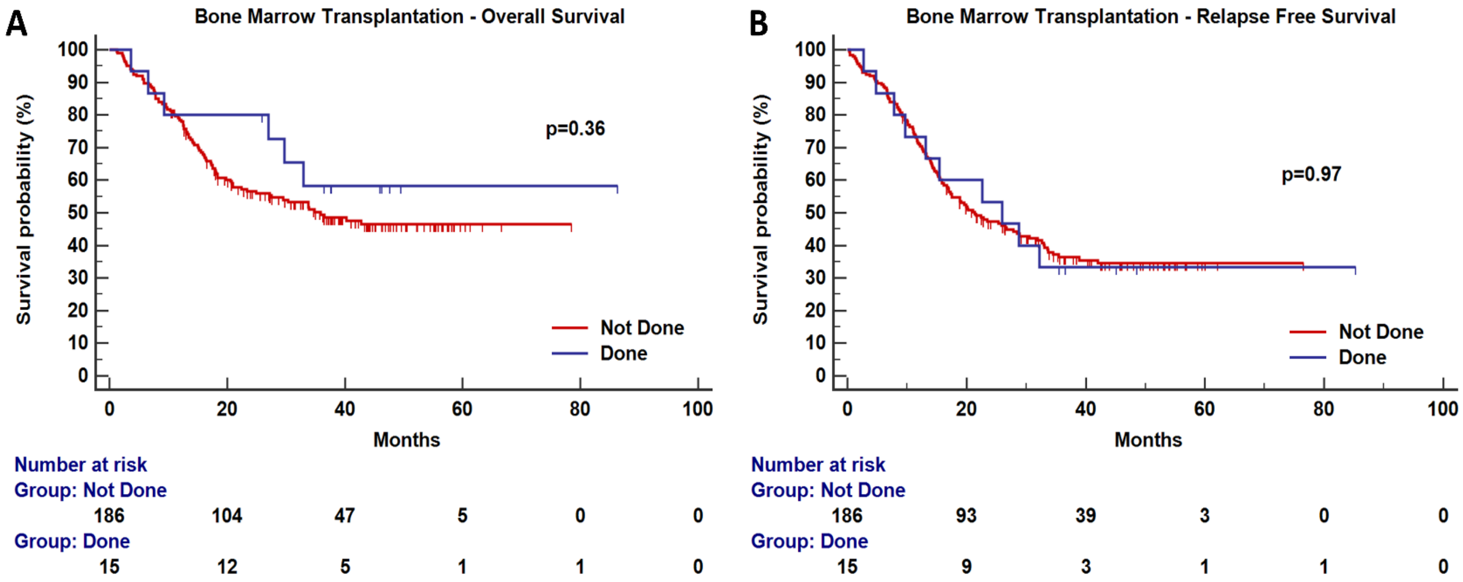

Supplementary Figure 3: Survival estimates for overall survival (A) and relapse free survival (B) of allogeneic bone marrow transplantation in this patient cohort.

b. *Rebalancing smMIPS to ensure uniform capture:*

Following initial equimolar pooling and analysis of read depths, the smMIPS pool underwent two rounds of rebalancing. A balanced version can be seen in supplementary Figure 4.

Supplementary Methods

Patkar et al. Molecular MRD Detection in AML using Error Corrected NGS

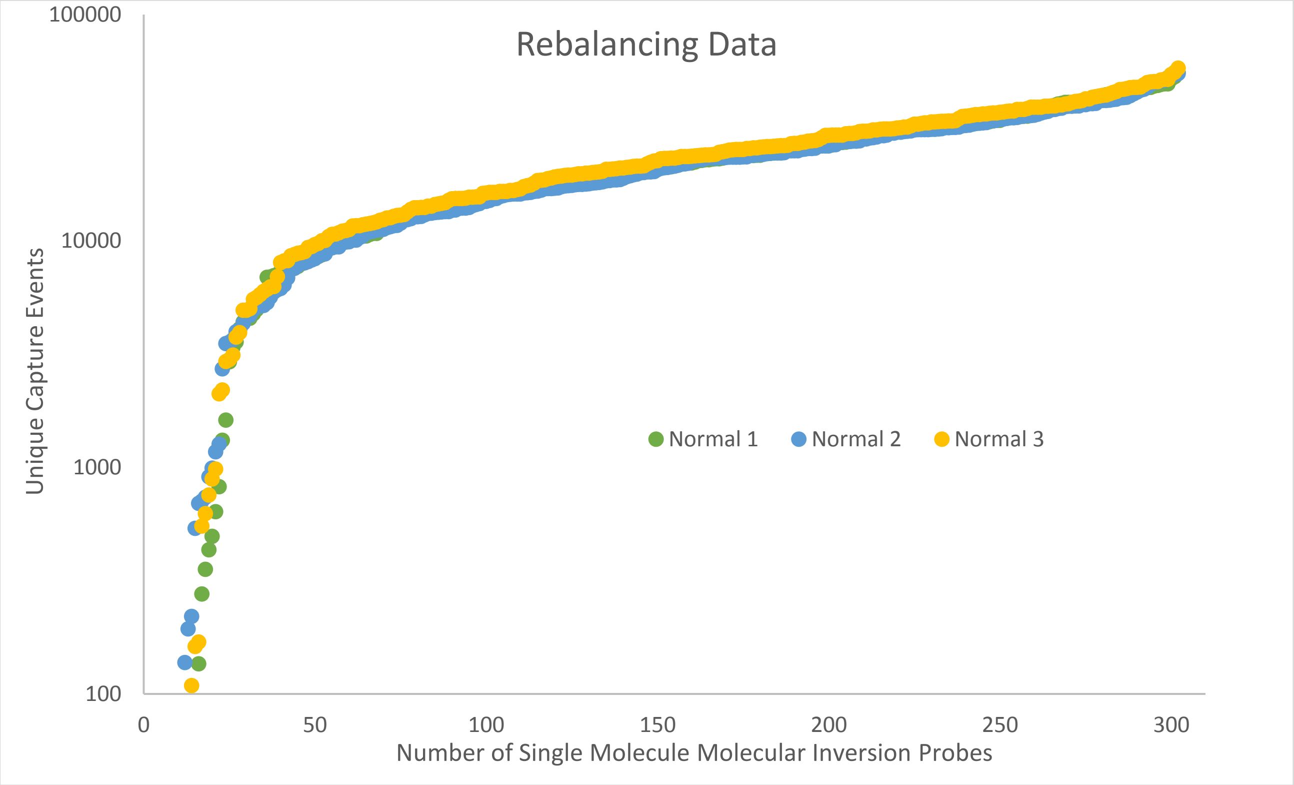

Supplementary Figure 4: Unique Capture Events per smMIP for three normal specimens.

a. *Sequencing:*

Initial standardization and balancing experiments using NA12878 control DNA were performed on a MiSeq (standard V2 flow cell 150PE chemistry). Once the assay was standardized, error modelling, limit of detection and MRD detection experiments were performed on multiple S4 flow cells of a NovaSeq 6000 using 150PE chemistry.

b. *Data Analysis:*

The bioinformatics approach was similar to that published by Waalkes et al[2] with a few modifications. Demultiplexing was performed using bcl2fastq-v2.17. Adapter sequences, smMIPs backbone and reads less than 53bp were trimmed using fastq-mcf tool of ea-utils (https://expressionanalysis.github.io/ea-utils/). Paired end assembly was carried out using PEAR (v0.9.8).[3] A custom script was used to trim, concatenate the 4bp UMI at the 5' and 3' end of assembled read and add it to the read header. Reads were mapped to the human genome (build hg19) using bwa-0.7.12[4] and pre-processed using SAMtools-1.1[5]. For computational efficiency, mapped reads were split by chromosome and into files sourced from reads mapping to individual smMIPS based on their genomic start and stop coordinates. Each of these files is processed individually for downstream variant calling. All reads originating from a single UMI were discarded (singleton reads). The rest of the reads (two or more UMI) were used to create a consensus .sam file using CallMolecularConsensusReads function of fgbio-0.4.0 with the following parameters --error-rate-post-umi=30 --min-reads=2 --min-input-base-quality 20 (https://github.com/fulcrumgenomics/fgbio). These reads were converted to fastq using picard-2.17.2 (http://broadinstitute.github.io/picard/) and mapped, sorted and indexed using bwa and samtools as described above. A .mpileup was created using samtools-1.1 and sequence variants detected using a bespoke variant caller.

(https://bitbucket.org/uwlabmed/smmips_analysis/src/master/). Variant calls from individual smMIPs were combined into a single .vcf file and annotated using ANNOVAR.[6]

AML NGS MRD Panel			
**Sr. Nos**	**Gene Name**	**Region**	**RefSeqID**
1	***ATM***	Exon **37**	NM_000051
2	*BCOR*	Exon 4, 10, 11	NM_001123383
3	*DNMT3A*	Exon 23	NM_022552
4	*EZH2*	Exon 14-18	NM_152998.2
5	*FLT3*	Exon 20, 14-15*	NM_004119
6	*GATA1*	Exon 2	NM_002049
7	*GATA2*	Exon 3, 4	NM_032638
8	*IDH1*	Exon 4	NM_005896
9	***IDH2***	Exon 4, Exon 4 **(R172K)**	NM_002168
10	*JAK2*	Exon 14	NM_004972
11	*KDM6A*	Exon 17, 20	NM_021140
12	*KIT*	Exon 8-11, 17	NM_000222
13	***KMT2D***	Exon **34, 39**	NM_003482
14	***KRAS***	Exon 2-4, **5**	NM_004985
15	*NF1*	Exon 24, 47	NM_001042492
16	***NOTCH1***	Exon **27, 34**	NM_017617
17	***NOTCH2***	Exon **34**	NM_024408
18	*NPM1*	Exon 11	NM_002520
19	*NRAS*	Exon 2-3	NM_002524
20	*PHF6*	Exon **2**, 3-9	NM_032458
21	*PTPN11*	Exon 3, 13-14	NM_002834
22	***RAD21***	Exon **5, 11**	NM_006265
23	*RUNX1*	Exon 4-8	NM_001754
24	***SETBP1***	Exon **4**	NM_015559
25	*SF3B1*	Exon 14-16	NM_012433
26	***SH2B3***	Exon **3**, 6	NM_005475
27	***SMC1A***	Exon **2, 11**	NM_006306
28	*SRSF2*	Exon 1	NM_003016
29	*STAG2*	Exon 7-8, 19, 24, 27-29	NM_006603
30	*TET2*	Exon 3-11	NM_001127208
31	*TP53*	All Exons	NM_001126114
32	*U2AF1*	Exon 2, 6, **7**	NM_006758
33	*WT1*	Exon 6-8, 11	NM_024426
34	*ZRSR2*	Exon 10	NM_005089

Supplementary Table 1: List of genes and their loci sequenced using the smMIPS AML MRD panel. **FLT3*-ITD MRD was performed using a one-step PCR based ultradeep sequencing assay

	**Favourable Risk**		**Intermediate Risk**		**Poor Risk**	
	Total Number of Patients	Mutations in DTA Genes	Total Number of Patients	Mutations in DTA Genes	Total Number of Patients	Mutations in DTA Genes
**1 Mutation**	26	2	32	1	7	0
**2 Mutations**	13	0	33	5	3	0
**3 Mutations**	9	1	48	11	6	0
**4 Mutations**	0	0	18	9	1	0
**>5 Mutations**	0	0	5	4	0	0

Supplementary Table 2: Applicability of NGS-MRD panel in AML patients stratified by cytogenetic risk and number of mutations seen in DTA genes

c. *Limit of Detection Experiment:*

In order to determine the limit of detection, we performed an experiment where OCIAML3 cell line [harbouring *DNMT3A* (p.R882C), *NRAS* (p.Q61L) and *NPM1* (Type A mutation)] was serially diluted in a normal bone marrow (BM). Similarly, baseline DNA from five AML samples [each harbouring *NRAS* (p.G12D), *IDH1* (p.R132G), *IDH2* (p.R140Q), *IDH2* (p.R172K) and *NPM1*

(type A)] were serially diluted in normal BM as seen in Supplementary Figure 5. The expected variant allele frequency (VAF) for that mutation was calculated from the original VAF found in undiluted sample. The range of the expected VAFs was from 1.25% to 0.02%. We could successfully detect VAFs in nearly all cases up to a lower limit of approximately 0.05%. The limit of detection of NGS-MRD assay was thus at 0.05% for all mutations and 0.03% for the *NPM1* mutation. A lower LOD threshold was acceptable for *NPM1* mutation because of the uniqueness of the indel mutation and a previous observation (based on a limit of blank study) that complex 4bp indels (seen in *NPM1*) are not observed for short read sequencing data.[7]

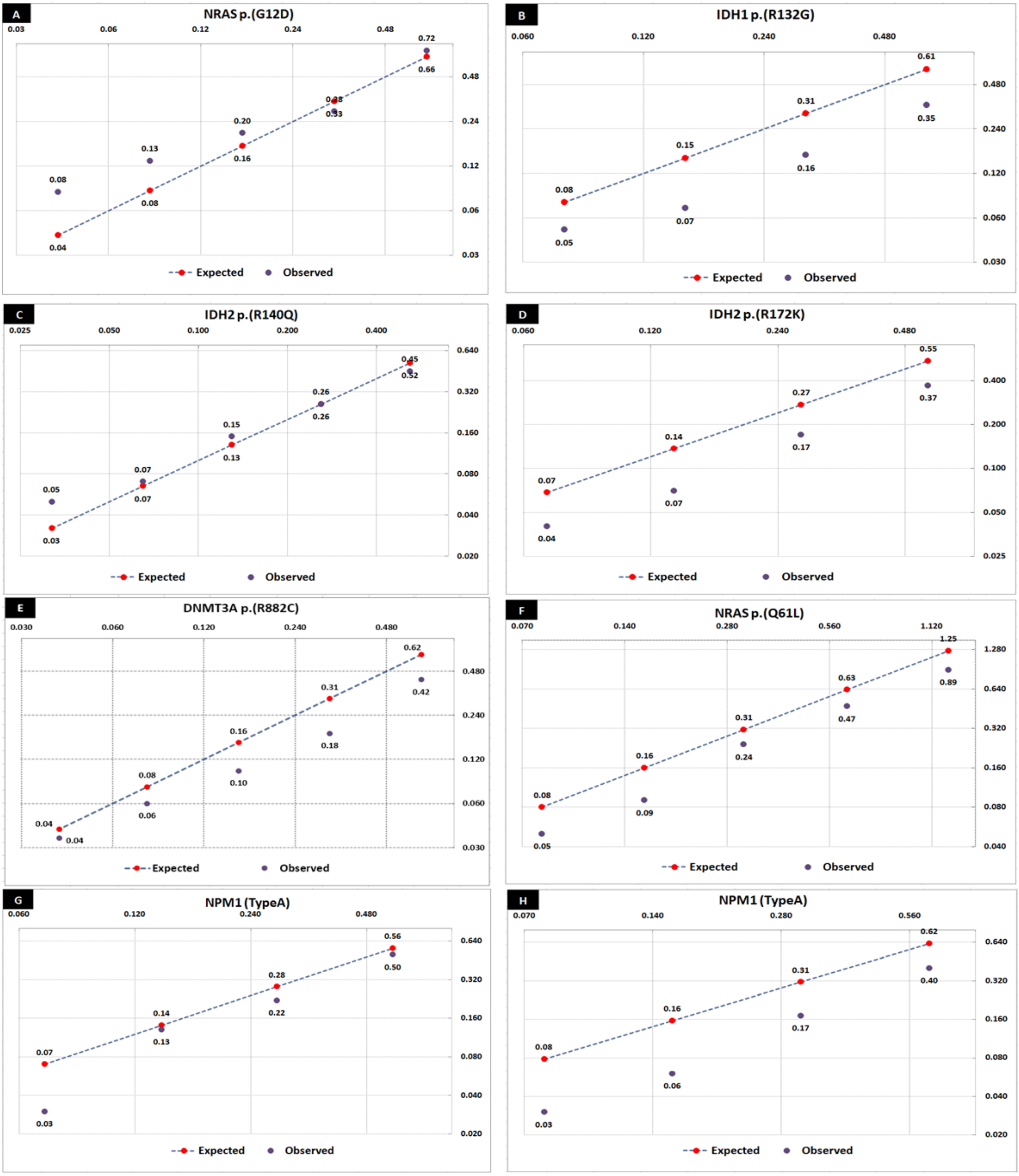

Supplementary Figure 5: Serial dilutions of OCIAML3 and AML DNA in normal bone marrow. The expected VAF for a mutation was calculated from the original VAF found in undiluted samples.

d. Error Modelling:

A site and mutation specific error model was setup to ascertain the occurrence of variations observed in the smMIP MRD panel. We sequenced a NA12878 and four normal bone marrow controls to measure sequencing errors using smMIPS as described by Waalkes and colleagues.[2] As mentioned by Waalkes we discovered a reduction in error rates using UMI based sequencing over standard NGS based sequencing (data not shown). As described, we fitted a β distribution for each base position and probable base substitution error. We observed a higher frequency of C>T and G>A changes consistent with oxidative DNA damage (Supplementary Figure 6) occurring in template DNA before sequencing. Where no variation was detected a 1:15,000 error rate was presumed and a β distribution was modelled. For each variation observed, a site-specific p-value was annotated using these pre-calculated β distributions. Sites with p>0.005 were excluded as artefacts.

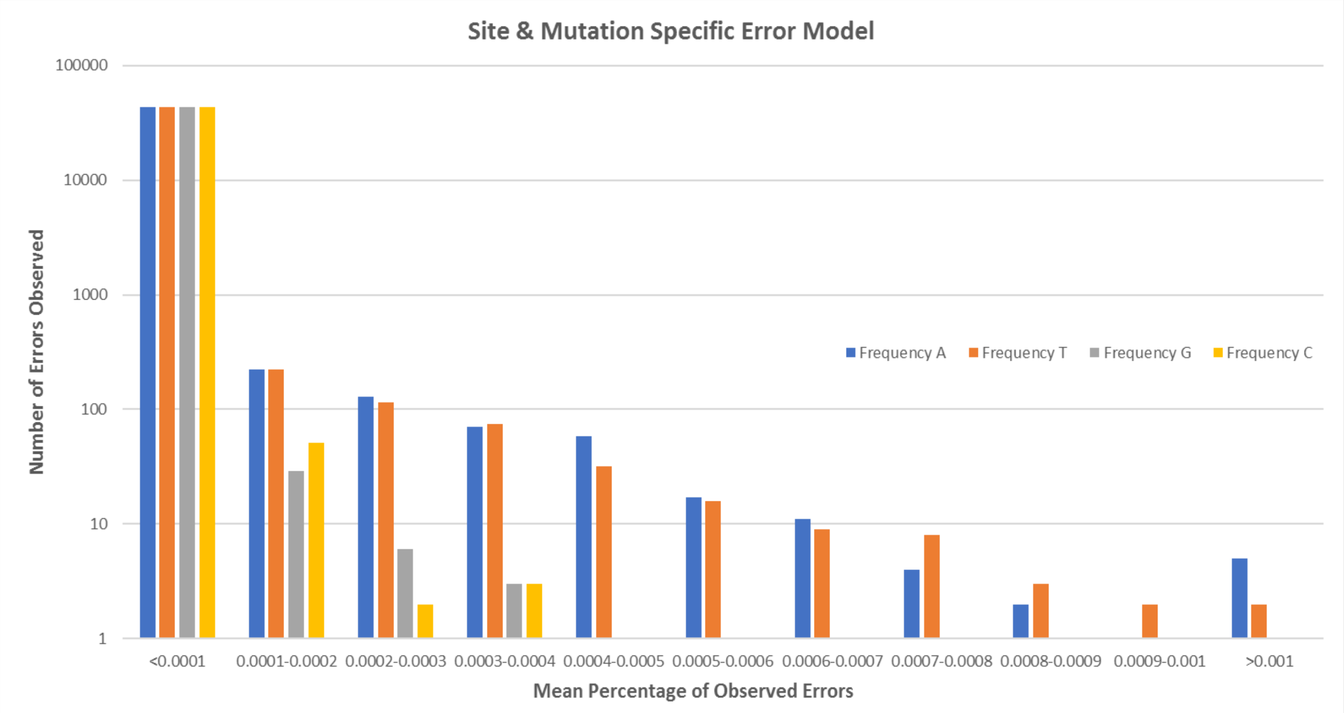

Supplementary Figure 6: Frequencies of errors observed using a site and mutation specific model.

e. Criteria for variant calling using smMIPS MRD assay:

i. Variants filtered by focussing on exonic regions (including splicing variants if any) followed by population frequency (<0.01) filtering.

ii. The variant must have been detected at baseline.

iii. Background error modelling at that site must have a *P* value <0.005.

iv. A minimum of 10 alternate variant reads must be present for an SNV.

v. A minimum of 3 alternate variant reads must be present for an indel.

vi. The highest VAF was taken as MRD.

vii. If the highest VAF was in a variant associated with *DNMT3A, TET2* or *ASXL1* (DTA mutation) it was ignored.

Supplementary Methods

Patkar et al. Molecular MRD Detection in AML using Error Corrected NGS

2. Detection of *FLT3*-MRD using a one-step PCR assay:

a. Assay Design:

We observed that we could not monitor *FLT3*-ITD using smMIPs. To overcome this issue, we designed a one-step PCR assay that incorporated locus specific primers, dual indices and Illumina adapters in a single step (Supplementary Table 3). The primers were designed to amplify common internal tandem duplication site (chr13: 28608024 - 28608353).[8] The assay was setup using 600ng of genomic DNA as template. The below primers (50nM each) were added to 12.5μL of HotStarTaq Master Mix (Qiagen, Hilden, Germany) to setup a 25 μL reaction. Amplification was carried out under the following conditions; (Denaturation: 95°C-15 minutes; Denaturation: 95°C-60 seconds; Annealing 61°C-60 seconds; Extension 72°C-60 seconds; 35 cycles; final extension 72°C-45 seconds). The final library was size selected using Agencourt AMPure XP beads (Beckman Coulter Inc., California, USA) and sequenced on an Illumina MiSeq (V2, 250PE chemistry). Each sample was allotted 1.1 million reads.

***FLT3-ITD* primers**	**Sequences**
Forward Primer*	5'AATGATACGGCGACCACCGAGATCTACAC**XXXXXXXXXX**TATGGTGCCTGTAGCAATTTAGGTATGAAAGCCAGCTA
Reverse primer*	5'CAAGCAGAAGACGGCATACGAGAT**XXXXXXXXXX**AGTCAGTCAGTCCTTTCAGCATTTTGACGGCAACC
Read 1 Primer	5'- TATGGTGCCTGTAGCAATTTAGGTATGAAAGCCAGCTA-3'
Read 2 primer	5' AGTCAGTCAGTCCTTTCAGCATTTTGACGGCAACC-3'
Index Primer	5'- GGTTGCCGTCAAAATGCTGAAAGGACTGACTGACT-3'

Supplementary Table 3: Adapter tagged *FLT3*-ITD primers *- XXXXXXXXXX represents 10 bp sample specific index sequence.

b. Data Analysis and Limit of Detection:

We adopted a recently described algorithm[9] for accurate detection of *FLT3*-ITD using next generation sequencing. We could demonstrate good correlation (Supplementary Figure 7, inset) between conventional and NGS testing for accurate detection of ITD length in 71 *FLT3*-ITD positive AML. We could validate this assay till a maximum ITD length of 100bp.

c. *Limit of Detection of FLT3 NGS MRD assay*:

In order to determine the limit of detection of this assay, we diluted a *FLT3*-ITD (30bp ITD) positive sample into normal BM. All dilutions were performed in triplicates (Supplementary Figure 7). We could successfully detect this mutation till a lower limit of 0.002% VAF, as shown in Supplementary Figure 7. Based on this data, we established the limit of detection of the *FLT3*-ITD NGS MRD assay at 0.002. All FLT3-ITD clones >1% VAF were tracked

Supplementary Methods

Patkar et al. Molecular MRD Detection in AML using Error Corrected NGS

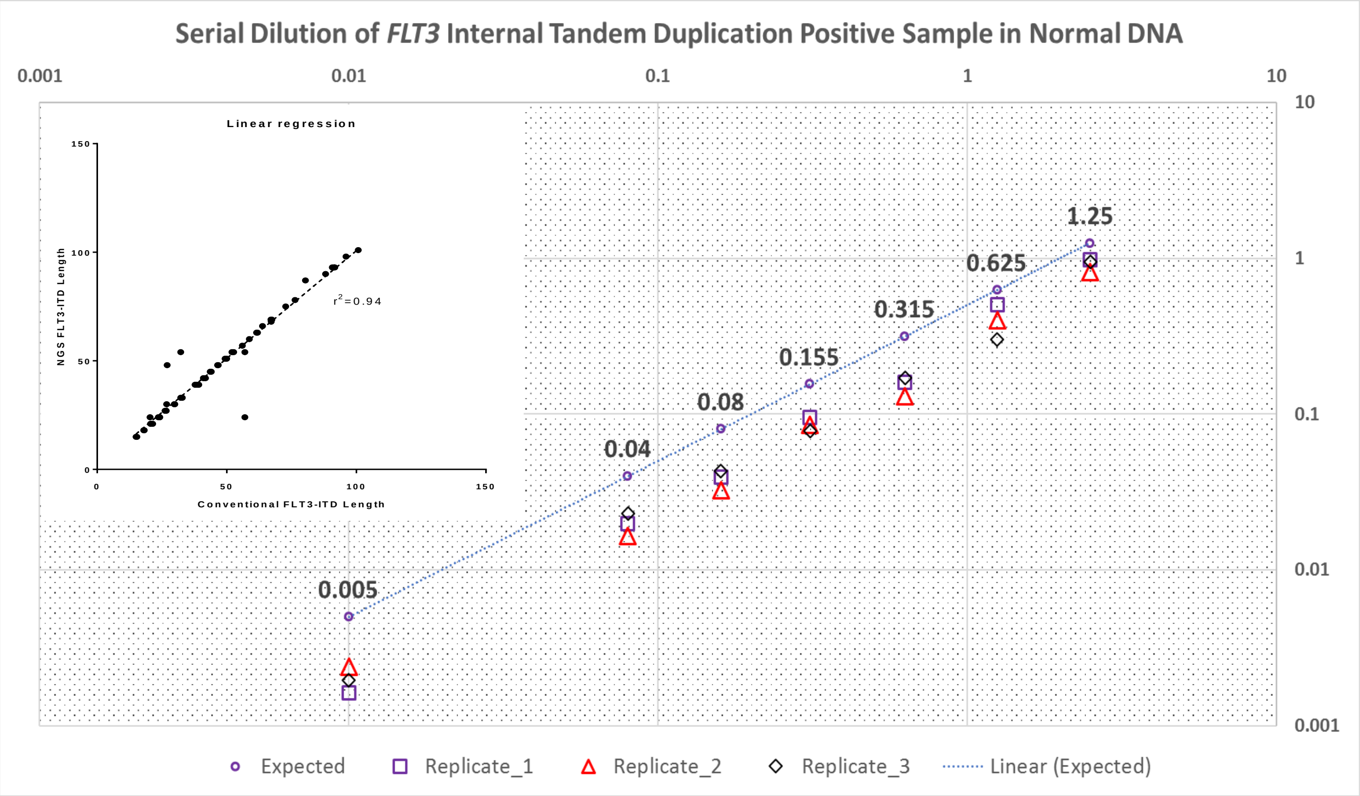

Supplementary Figure 7: Correlation of *FLT3*-ITD length by capillary (conventional testing) electrophoresis as compared to NGS assay (Inset). Serial dilution of a *FLT3*-ITD positive sample in normal DNA demonstrates the limit of detection (in triplicates) to be 0.002%.

Cytogenetic Risk	Post Induction FCM MRD		Post Induction NGS MRD	
	MRD Positive	MRD Negative	MRD Positive	MRD Negative
**Favorable**	21 (23.86%)	27 (24.11%)	30 (21.58%)	17 (29.82%)
**Intermediate**	55 (62.50%)	80 (71.43%)	99 (71.22%)	34 (59.65%)
**Poor Risk**	12 (13.64%)	5 (4.46%)	10 (7.19%)	6 (10.53%)
**Total**	88	112	139	57

Supplementary Table 4: Distribution of MRD results measured by FCM and NGS into cytogenetic risk categories. FCM: Flow Cytometry; NGS: Next generation sequencing. Chi Squared test did not reveal a statistical significance of MRD results distributed by cytogenetic risk groups.

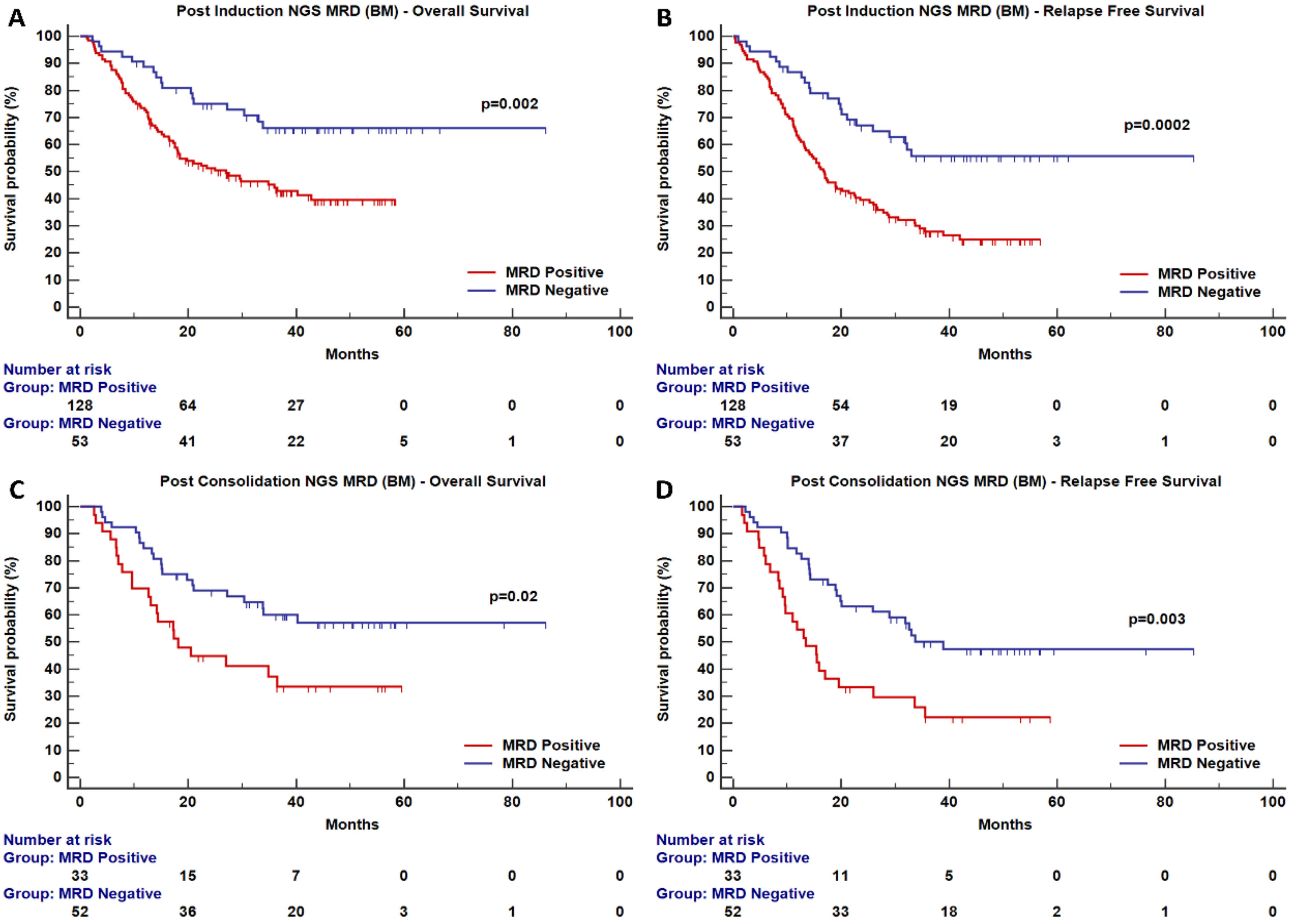

Supplementary Figure 8: Survival analysis for end of induction and post consolidation using NGS-MRD (After excluding samples sourced from blood).

Patkar et al. Molecular MRD Detection in AML using Error Corrected NGS

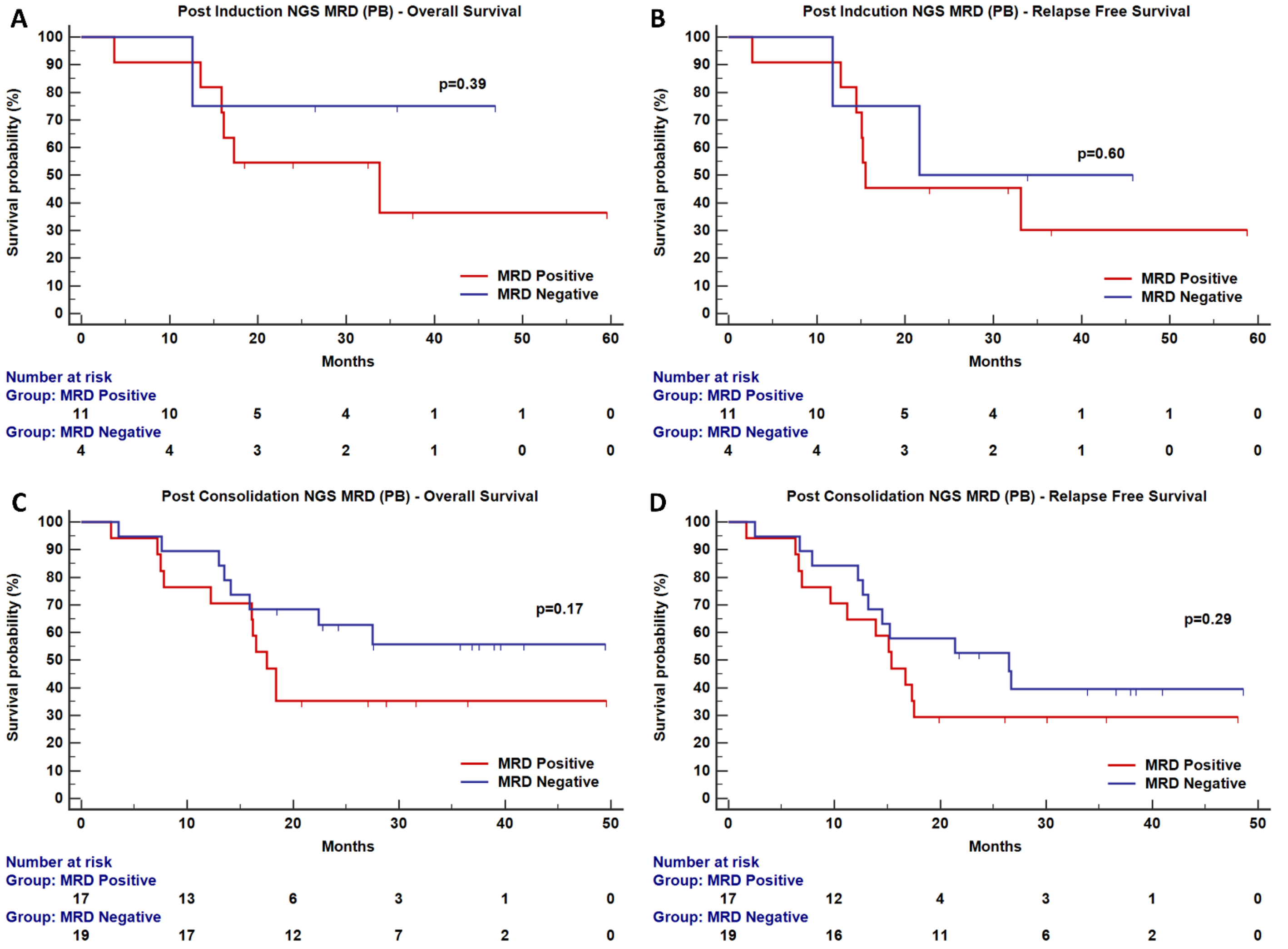

Supplementary Figure 9: Survival analysis for end of induction and post consolidation using NGS-MRD (After excluding samples sourced from bone marrow).

Supplementary Methods

Patkar et al. Molecular MRD Detection in AML using Error Corrected NGS

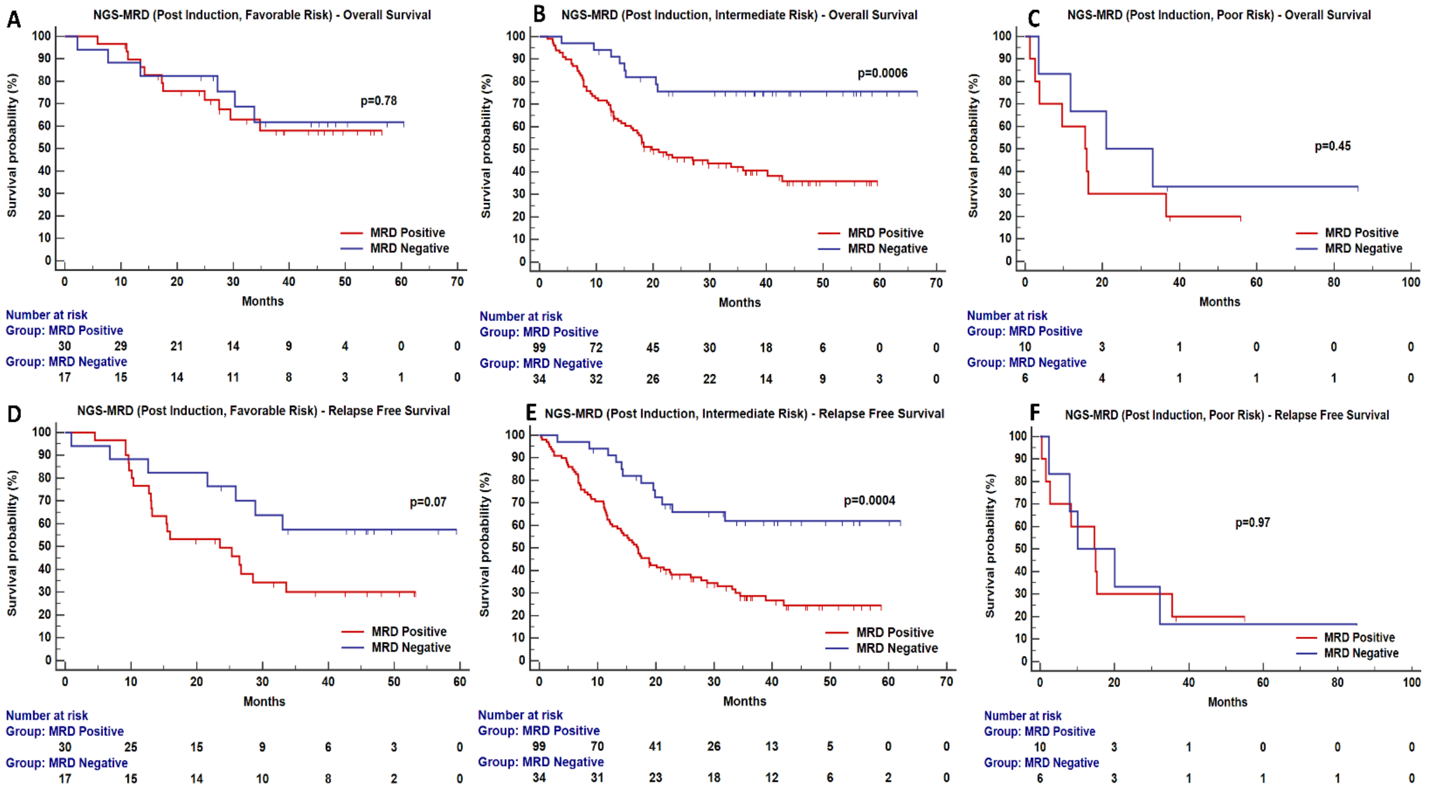

Supplementary Figure 10: Clinical relevance of NGS-MRD and their influence on overall survival (OS) and relapse free survival (RFS) when patients are grouped by cytogenetic risk categories.

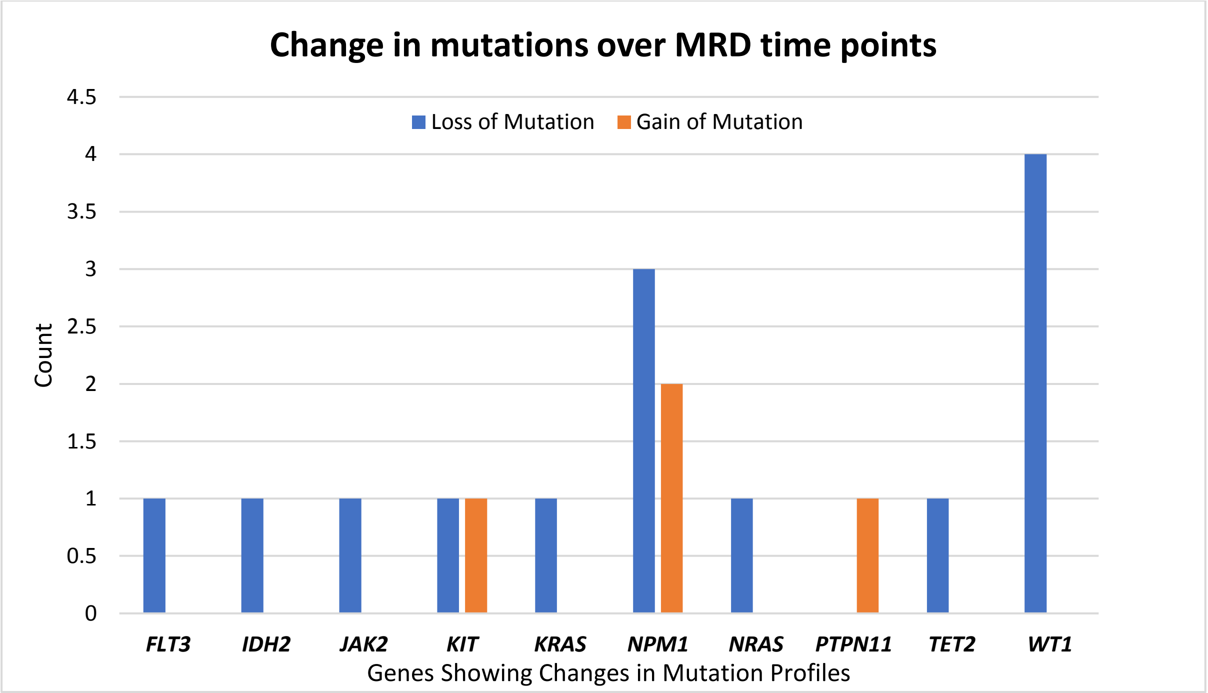

Supplementary Figure 11: Change in mutations over MRD time points amongst paired PI and PC samples.

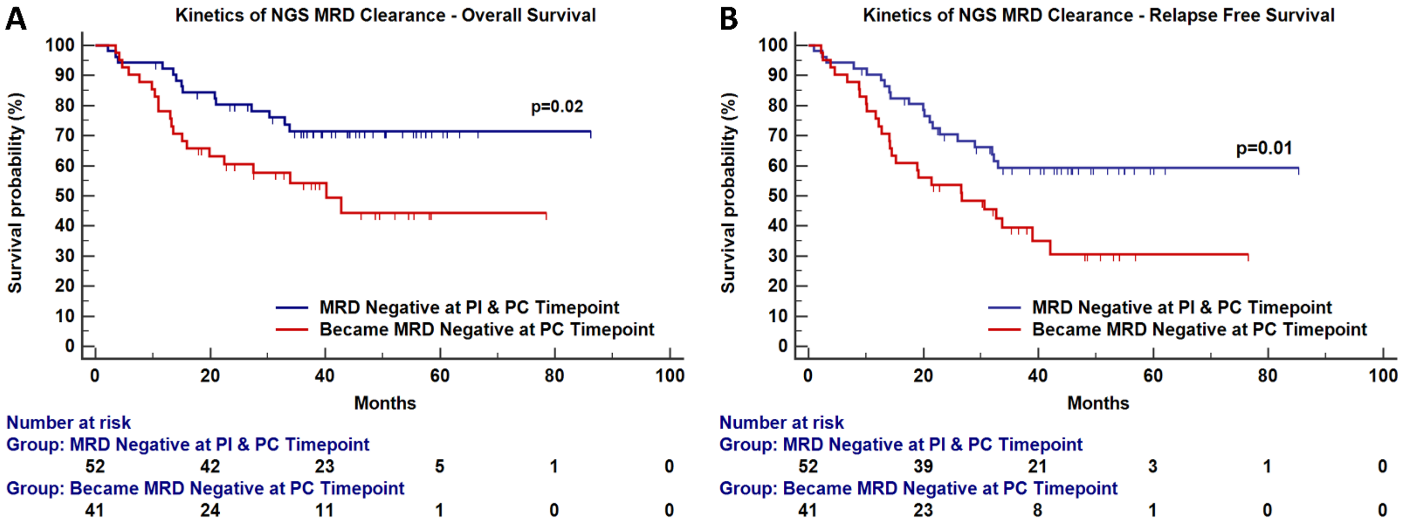

Supplementary Figure 12: Clinical relevance of kinetics of NGS-MRD clearance and their influence on overall survival (OS) and relapse free survival (RFS).

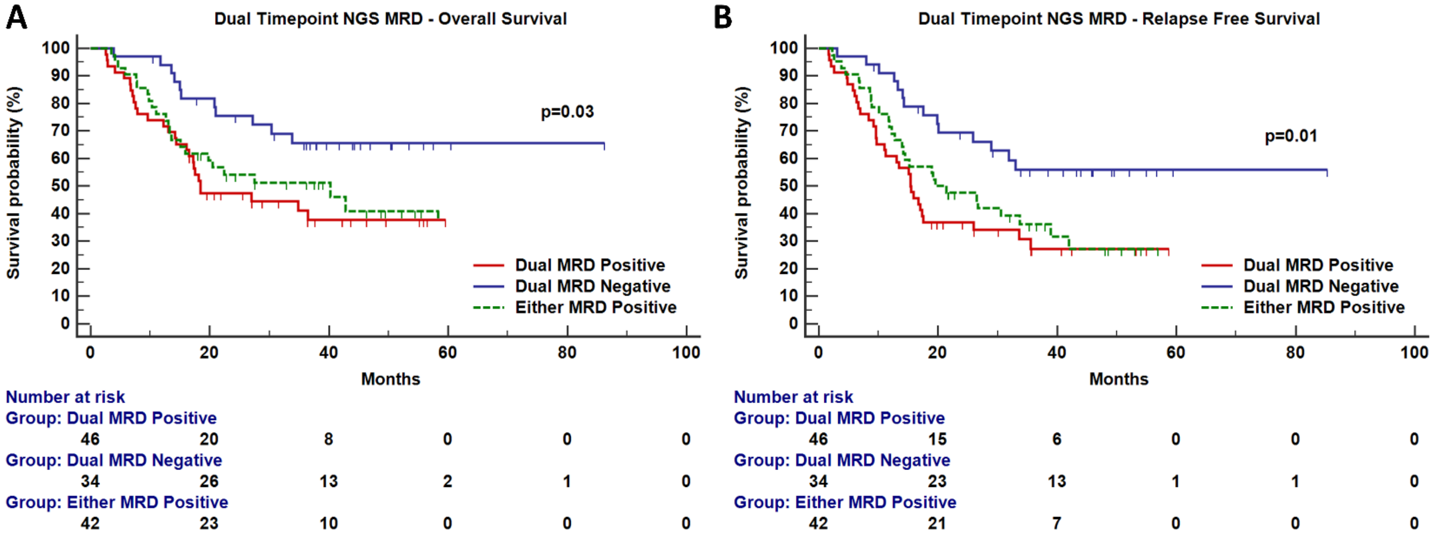

Supplementary Figure 13: Clinical relevance paired MRD sampling and influence on overall survival (OS) and relapse free survival (RFS). Patients who persistently harboured MRD had a significantly inferior OS [HR- 2.48; 95% CI- 1.33 to 4.61; (p=0.03)] and RFS [HR- 2.55; 95% CI- 1.45 to 4.48; (p=0.01)] as compared to patients who were MRD negative at both time points.

Dual Timepoint NGS MRD	Overall Survival (OS)		Relapse Free Survival (RFS)	
	HR (95% CI)	P	HR (95% CI)	P
MRD Negative	1	***0.03***	1	***0.01***
Either MRD Positive	1.2 (0.66 to 2.28)		1.2 (0.71 to 2.21)	
MRD Positive	2.5 (1.33 to 4.61)		2.5 (1.45 to 4.48)	
**Dual Timepoint NGS MRD**	**Overall Survival (OS)**		**Relapse Free Survival (RFS)**	
	**HR (95% CI)**	**P**	**HR (95% CI)**	**P**
MRD Negative	Mean OS: 63.2 months; 95% CI (52.0 to 74.4 months), Median OS: Not Reached	***0.03***	Mean OS: 55.8 months; 95% CI (44.1 to 67.5 months), Median OS: Not Reached	***0.01***
Either MRD Positive	Mean OS: 34.4 months; 95% CI (27.4 to 41.4 months), Median OS: 40.2 months; 95% CI (15.1 to 42.8 months)		Mean OS: 28.4 months; 95% CI (22.1 to 34.6 months), Median OS: 19.6 months; 95% CI (14.0 to 38.9 months)	
MRD Positive	Mean OS: 31.5 months; 95% CI (24.6 to 38.4 months), Median OS: 18.4 months; 95% CI (16.1 to 36.5 months)		Mean OS: 25.4 months; 95% CI (18.9 to 31.9 months), Median OS: 15.4 months; 95% CI (11.0 to 26.0 months)	
**Comparative Analysis of FCM MRD and NGS MRD (Post Induction)**	**Overall Survival (OS)**		**Relapse Free Survival (RFS)**	
	**HR (95% CI)**	**P**	**HR (95% CI)**	**P**
NGS MRD- FCM MRD-	1	***0.0002***	1	***0.0001***
NGS MRD+ FCM MRD-	1.6 (0.96 to 2.61)		1.4 (0.87 to 2.12)	
NGS MRD- FCM MRD+	1.3 (0.58 to 2.74)		1.3 (0.63 to 2.52)	
NGS MRD+ FCM MRD+	4.7 (2.71 to 8.00)		4.0 (2.51 to 6.47)	
**Comparative Analysis of FCM MRD and NGS MRD (Post Induction)**	**Overall Survival (OS)**		**Relapse Free Survival (RFS)**	
	**HR (95% CI)**	**P**	**HR (95% CI)**	**P**
NGS MRD- FCM MRD-	Mean OS: 71.5 months; 95% CI (62.4 to 80.7 months), Median OS: Not Reached	***0.0002***	Mean OS: 63.7 months; 95% CI (53.5 to 73.8 months), Median OS: Not Reached	***0.0001***
NGS MRD+ FCM MRD-	Mean OS: 37.7 months; 95% CI (31.8 to 43.5 months), Median OS: 42.8 months; 95% CI (19.8 to 42.8 months)		Mean OS: 29.2 months; 95% CI (23.8 to 34.5 months), Median OS: 19.1 months; 95% CI (14.9 to 33.1 months)	
NGS MRD- FCM MRD+	Mean OS: 32.7 months; 95% CI (22.2 to 43.0 months), Median OS: 21.0 months; 95% CI (14.1 to 33.8 months)		Mean OS: 27.0 months; 95% CI (17.5 to 36.5 months), Median OS: 19.6 months; 95% CI (11.8 to 33.0 months)	
NGS MRD+ FCM MRD+	Mean OS: 28.7 months; 95% CI (23.4 to 34.0 months), Median OS: 17.5 months; 95% CI (13.5 to 35.9 months)		Mean OS: 18.4 months; 95% CI (18.4 to 27.7 months), Median OS: 15.4 months; 95% CI (11.7 to 18.9 months)	
**Comparative Analysis of FCM MRD and NGS MRD (Post Consolidation)**	**Overall Survival (OS)**		**Relapse Free Survival (RFS)**	
	**HR (95% CI)**	**P**	**HR (95% CI)**	**P**

NGS MRD- FCM MRD-	1	***0.02***	1	***0.001***
NGS MRD+ FCM MRD-	2.2 (0.60 to 7.83)		2.2 (0.64 to 7.65)	
NGS MRD- FCM MRD+	2.0 (0.43 to 9.34)		1.6 (0.35 to 7.02)	
NGS MRD+ FCM MRD+	3.7 (1.07 to 13.0)		4.1 (1.27 to 13.70)	
**Comparative Analysis of FCM MRD and NGS MRD (Post Consolidation)**	**Overall Survival (OS)**		**Relapse Free Survival (RFS)**	
	**HR (95% CI)**	**P**	**HR (95% CI)**	**P**
NGS MRD- FCM MRD-	Mean OS: 33.5 months; 95% CI (29.2 to 37.8 months), Median OS: Not Reached	***0.02***	Mean OS: 30.9 months; 95% CI (26.6 to 35.1 months), Median OS: 38.9 months; 95% CI (26.5 to 38.9 months)	***0.001***
NGS MRD+ FCM MRD-	Mean OS: 34.6 months; 95% CI (26.1 to 43.2 months), Median OS: 20.5 months; 95% CI (17.3 to 36.5 months)		Mean OS: 28.3 months; 95% CI (20.1 to 36.4 months), Median OS: 16.7 months; 95% CI (15.1 to 35.5 months)	
NGS MRD- FCM MRD+	Mean OS: 43.9 months; 95% CI (20.7 to 367.1 months), Median OS: 21.0 months; 95% CI (11.7 to 33.9 months)		Mean OS: 24.9 months; 95% CI (8.3 to 41.5 months), Median OS: 14.1 months; 95% CI (10.1 to 32.6 months)	
NGS MRD+ FCM MRD+	Mean OS: 18.2 months; 95% CI (8.04 to 28.4 months), Median OS: 12.6 months; 95% CI (5.60to 27.0 months)		Mean OS: 13.4 months; 95% CI (5.1 to 21.8 months), Median OS: 9.6 months; 95% CI (4.7 to 15.4 months)	

Supplementary Table 5: Comparative analysis between the two modalities FCM-MRD and NGS-MRD at post induction and post consolidation timepoints. OS: Overall Survival, RFS: Relapse Free Survival, CI: confidence interval.

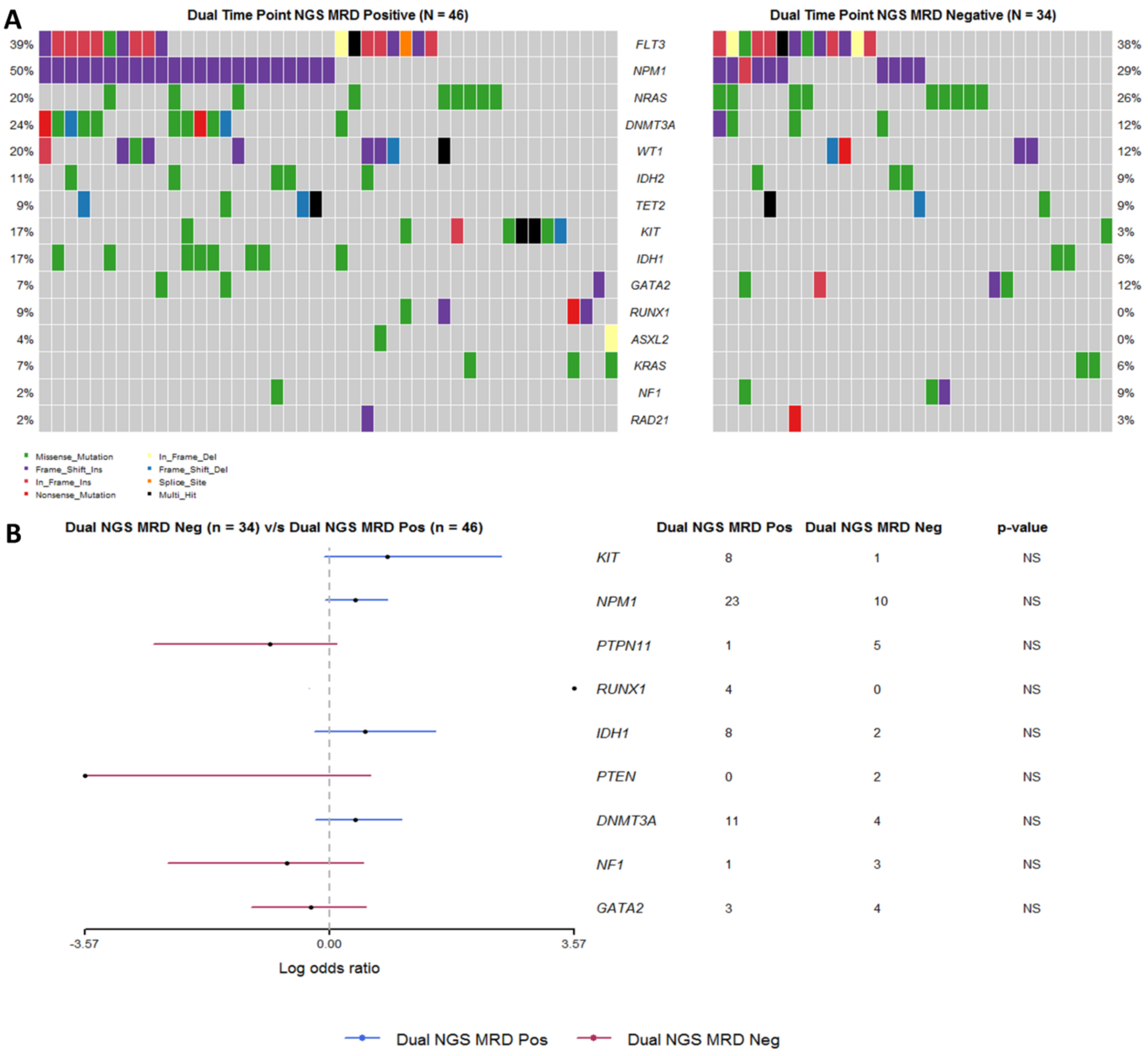

Supplementary Figure 14: Comparative oncoplot (A) of patients who were NGS-MRD positive at both time points as compared to patients who were persistently MRD negative. Commonly occurring mutations are highlighted here. Forest plot (B) fails to demonstrate a genetic difference between these two groups.

3. **<u>Orthogonal MRD detection of *NPM1* mutations using *NPM1* MRD assay</u>.**

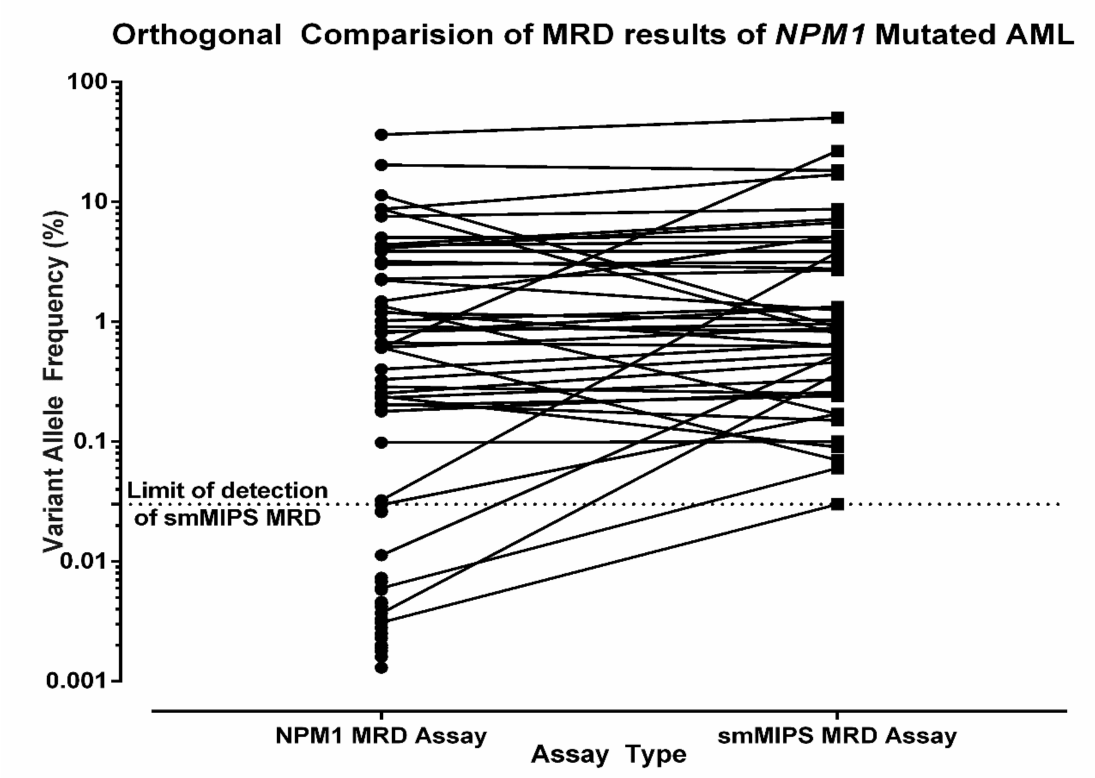

Supplementary Figure 15: Cross validation of smMIPS MRD cases (n=75; *NPM1* mutated AML) detected by *NPM1* NGS MRD assay. Since the axis is logarithmic, zero values (n=44) have been omitted.

Orthogonal MRD testing was performed in 75 MRD samples (75 out of 323 MRD samples; 23.2%). These were detected using a previously published ultradeep sequencing based MRD assay for *NPM1* mutated AML.[7] smMIPS MRD positive *NPM1* mutated AML cases (45 out of 75; 60%) could be detected by the *NPM1* NGS MRD assay. For the rest of the cases, *NPM1* mutation was either negative or detected at a threshold below the smMIPS MRD assay limit. For smMIPS MRD positive cases the median MRD value was 0.86% as compared to 0.82% for *NPM1* NGS MRD (at a LOD cut-off of 0.03%).

4. FCM based AML-MRD:

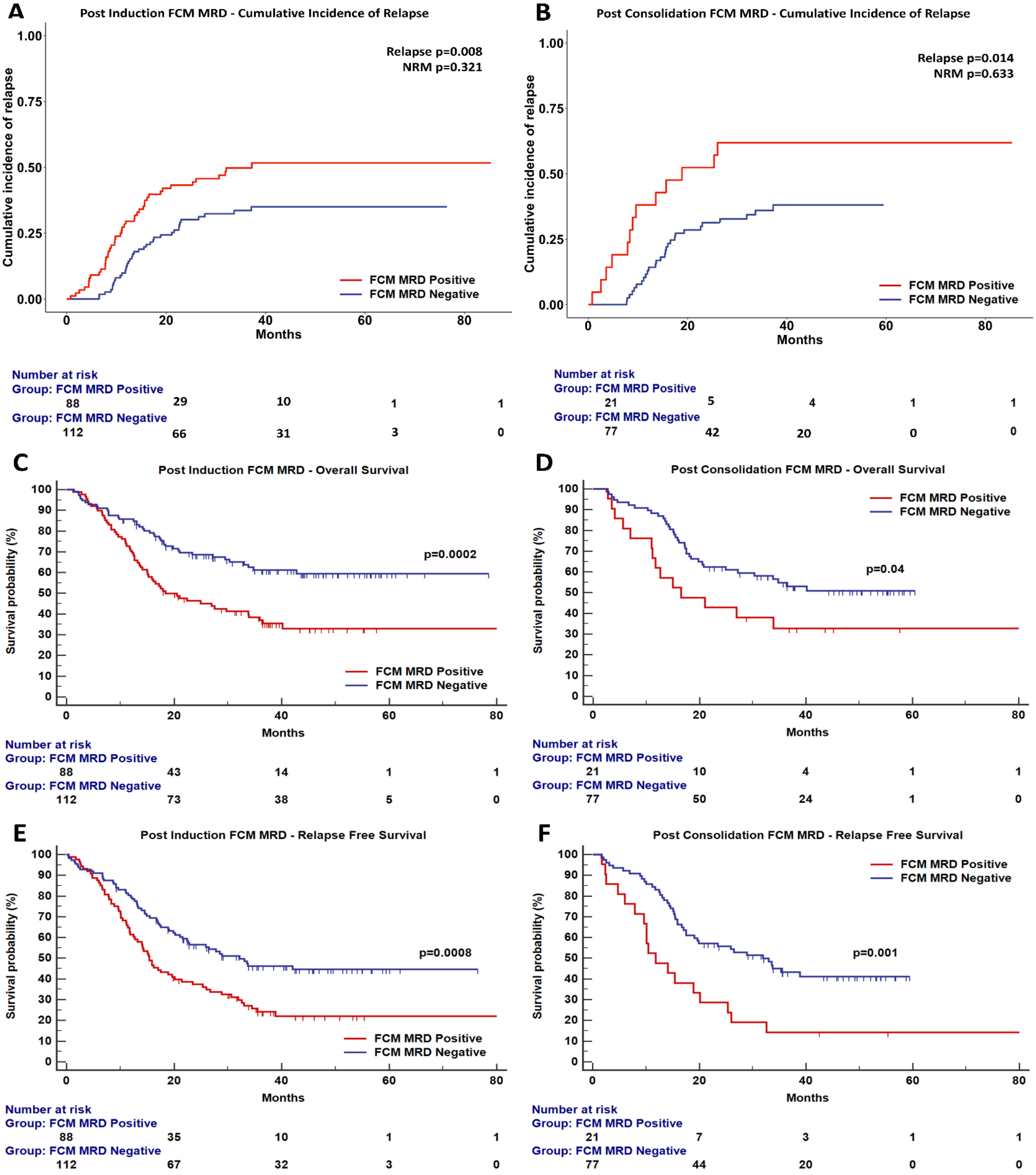

Supplementary Figure 16: Survival analysis of adult AML patients by flow cytometric MRD assessment (FCM-MRD) after induction and consolidation phases of chemotherapy.

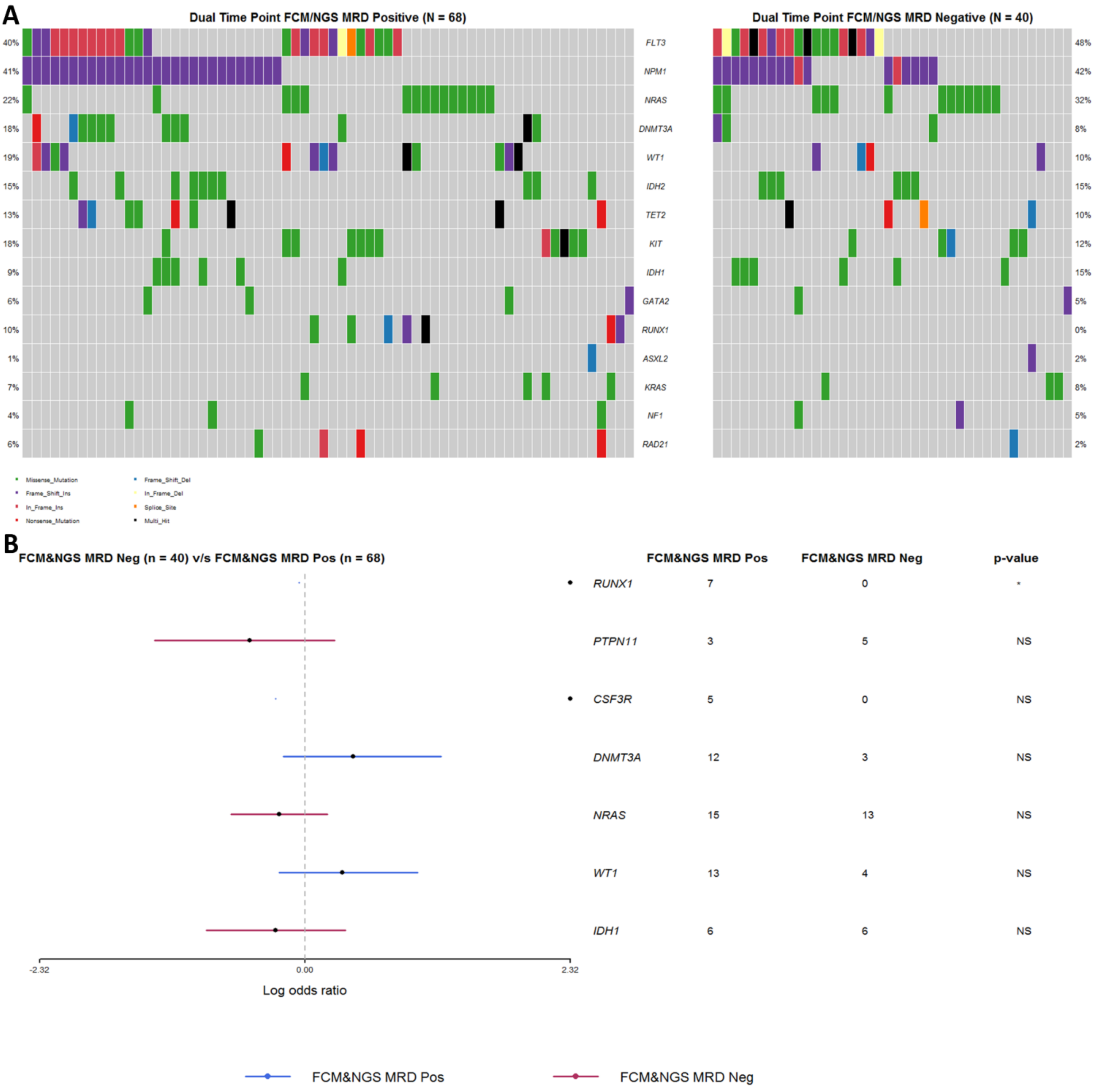

Supplementary Figure 17: Comparison of genetic profiles between FCM-MRD and NGS-MRD groups (FCM+NGS+ vs FCM-NGS-) at PI timepoint. Comparison is performed using Fischers exact test on all genes between two cohorts (*RUNX1* mutation, p=0.04).

Supplementary Methods

Patkar et al. Molecular MRD Detection in AML using Error Corrected NGS

UPN	Baseline Mutations	Number Covered by NGS Panel	Mutation_Details	FISH	Karyotyping	Cytogenetic Risk	PI NGS-MRD Consensus Coverage	PI NGS-MRD (VAF)	Post Induction NGS MRD	PC NGS-MRD Consensus Coverage	PC NGS-MRD (VAF)	Post Consolidation NGS MRD	Post Induction Flow MRD	Post Consolidation Flow MRD	Survival Status
Case 1	4	3	NRAS:NM_002524:exon2:c.G35A:p.G12D<br>KIT:NM_000222:exon17:c.G2446T:p.D816Y<br>WT1:NM_024426:exon7:c.C1142A:p.S381X<br>FLT3:NM_004119:exon16:c.C2028A:p.N676K	inv(16) and trisomy 8	Not available	Favorable	11427	NRAS- 0.5	Positive	18011		Negative	Positive	Positive	Relapsed
Case 2	1	1	KIT:NM_000222:exon17:c.G2446C:p.D816H	RUNX1-RUNX1T1 fusion: t(8;21)	Not available	Favorable		NA	NA	14314		Negative	Positive	Positive	Relapsed
Case 3	2	1	NRAS:NM_002524:exon2:c.G38T:p.G13V<br>ATRX:NM_000489:exon9:c.G1948A:p.E650K	RUNX1-RUNX1T1 fusion: t(v;8;21)	Not available	Favorable	9958		Negative	17653		Negative	Positive	Negative	Alive
Case 4	1	1	TET2:NM_001127208:exon6:c.G3799A:p.E1267K	RUNX1-RUNX1T1 fusion: t(8;21)	Not available	Favorable	20295		Negative	8716		Negative	Positive	Negative	Alive
Case 5	5	1	PTEN:NM_000314:exon7:c.697_697delinsGGG<br>DNMT3A:NM_022552:exon4:c.C446T:p.A149V<br>NRAS:NM_002524:exon3:c.A183C:p.Q61H<br>RAD21:NM_006265:exon8:c.C877T:p.Q293X	RUNX1-RUNX1T1 fusion: t(8;21)	46,XX, t(8;21)(q22;q22)[9] 46,XX[2]	Favorable	7271		Negative	8627		Negative	Positive	Negative	Died without Relapse
Case 6	1	1	U2AF1:NM_006758:exon2:c.C101A:p.S34Y	Negative	46,XY [20]	Intermediate	16126	U2AF1- 0.26	Positive	9788		Negative	Positive	Positive	Relapsed
Case 7	1	1	NRAS:NM_002524:exon2:c.G35A:p.G12D	Negative	46,XY[20]	Intermediate	16514		Negative		NA	NA	Positive	Negative	Alive
Case 8	1	1	BCOR:NM_001123383:exon4:c.G2503A:p.A835T	Negative	Not available	Intermediate	20553		Negative	19283		Negative	Positive	Positive	Relapsed
Case 9	1	1	FLT3-ITD	Negative	Not available	Intermediate	1151064 (FLT3-ITD)		Negative	1406146 (FLT3-ITD)		Negative	Positive	NA	Alive
Case 10	2	2	NRAS:NM_002524:exon2:c.G38A:p.G13D<br>FLT3-ITD	MLL translocation	Not available	Intermediate	15532		Negative	14215 , (512548 FLT3-ITD)	FLT3 ITD-0.006	Positive	Positive	Positive	Died without Relapse
Case 11	1	1	KRAS:NM_004985:exon4:c.G436A:p.A146T	Negative	Not available	Intermediate	14179		Negative	12020		Negative	Positive	NA	Relapsed
Case 12	3	3	TET2:NM_001127208:exon3:c.778_793AGTGTC<br>NPM1:NM_002520:exon11:c.859_860insTCTG:p.L287fs<br>PTPN11:NM_002834:exon13:c.G1530T:p.Q510H	Negative	Not available	Intermediate	13335		Negative	14160		Negative	Positive	Negative	Died without Relapse
Case 13	1	1	WT1:NM_024426:exon7:c.1109_1109delinsCC	Negative	46,XY, del(6)(q23.3)[3] 46,XY [7]	Intermediate	12200		Negative	14645		Negative	Positive	Negative	Relapsed
Case 14	2	2	NPM1:NM_002520:exon11:c.859_860insTCTG:p.L287fs<br>IDH2:NM_002168:exon4:c.G419A:p.R140Q	Negative	46,XX, ?inv(10) (p13;q22) [13] 46,XX [7]	Intermediate	8511		Negative	18301	NPM1- 0.42	Positive	Positive	Negative	Relapsed
Case 15	1	1	PTPN11:NM_002834:exon3:c.A172T:p.N58Y	Trisomy 8	47,XY,+8[13] 46,XY[2]	Intermediate	14838		Negative	22840		Negative	Positive	Negative	Died without Relapse
Case 16	2	1	NRAS:NM_002524:exon2:c.G38A:p.G13D<br>NF1:NM_001042492:exon34:c.A4462G:p.T1488A	MLL translocation: t(6;11)(q28;q23)	46,XY, t(6;11)(q27;q23)[3] 46,XY,t(6;11), t(9;11)(q22;q23)[4] 46,XY[3]	Poor	8652		Negative	10573		Negative	Positive	Positive	Relapsed
Case 17	3	3	IDH1:NM_005896:exon4:c.C394T:p.R132C<br>TP53:NM_001126114:exon5:c.G524A:p.R175H<br>U2AF1:NM_006758:exon6:c.G471C:p.Q157H	Negative	46,XX,del(X)(p11)[4], del(11)(q23)[6], del(12)(p13)[4], del(18)(p11)[2][cp9] 46,XX[3]	Poor	7925		Negative	14034		Negative	Positive	Positive	Relapsed
Case 18	3	3	SF3B1:NM_012433:exon15:c.A2098G:p.K700E<br>GATA2:NM_032638:exon4:c.A890G:p.N297S<br>KRAS:NM_004985:exon2:c.G35A:p.G12D	Monosomy 7	Not available	Poor	7281		Negative		NA	NA	Positive	Positive	Died without Relapse
Case 19	1	1	FLT3-ITD	Trisomy 5, 8 and 21	Not available	Poor	1469447 (FLT3-ITD)		Negative	1322385 (FLT3-ITD)		Negative	Positive	Positive	Relapsed
Case 20	1	1	GATA2:NM_032638:exon4:c.G952A:p.A318T	Negative	46,XX,del(5)(q33q35)[3] 46,XX, random abnormalities[2] 46,XX,[12]	Poor	1394		Negative	3917		Negative	Positive	Positive	Alive

Supplementary Table 6: Clinical, cytogenetic risk, molecular risk stratification, NGS MRD coverage, NGS MRD and Flow MRD results and outcome of patients who are FCM MRD positive but NGS MRD negative. NA: MRD not performed

Post Induction MRD Assessment	NGS+FCM+ vs NGS-FCM-		NGS+FCM- vs NGS-FCM-		NGS-FCM+ vs NGS-FCM-	
	**Value**	**95% CI**	**Value**	**95% CI**	**Value**	**95% CI**
Sensitivity	81.25%	69.54% to 89.92%	78.95%	66.11% to 88.62%	50.00%	29.12% to 70.88%
Specificity	63.64%	47.77% to 77.59%	52.83%	38.64% to 66.70%	84.85%	68.10% to 94.89%
Positive Predictive Value (*)	76.47%	68.36% to 83.02%	64.29%	56.78% to 71.15%	70.59%	49.36% to 85.53%
Negative Predictive Value (*)	70.00%	57.21% to 80.28%	70.00%	57.05% to 80.39%	70.00%	60.40% to 78.12%
Accuracy (*)	74.07%	64.75% to 82.03%	66.36%	56.73% to 75.09%	70.18%	56.60% to 81.57%
**Post Consolidation MRD Assessment**	**NGS+FCM+ vs NGS-FCM-**		**NGS+FCM- vs NGS-FCM-**		**NGS-FCM+ vs NGS-FCM-**	
	**Value**	**95% CI**	**Value**	**95% CI**	**Value**	**95% CI**
Sensitivity	28.57%	13.22% to 48.67%	48.72%	32.42% to 65.22%	25.93%	11.11% to 46.28%
Specificity	95.65%	78.05% to 99.89%	70.97%	51.96% to 85.78%	95.65%	78.05% to 99.89%
Positive Predictive Value (*)	88.89%	51.88% to 98.34%	67.86%	52.74% to 79.98%	87.50%	48.14% to 98.14%
Negative Predictive Value (*)	52.38%	46.14% to 58.55%	52.38%	42.93% to 61.66%	52.38%	46.40% to 58.29%
Accuracy (*)	58.82%	44.17% to 72.42%	58.57%	46.17% to 70.23%	58.00%	43.21% to 71.81%

Supplementary Table 7: Sensitivity, Specificity, PPV, NPV and Accuracy of both FCM-MRD and NGS-MRD assays.

	PI (NGS-MRD); n=15		PC (NGS-MRD); n=17	
	**NGS-MRD Positive**	**NGS-MRD Negative**	**NGS-MRD Positive**	**NGS-MRD Negative**
**FCM MRD Positive**	5	2	3	1
**FCM MRD Negative**	6	2	8	5

Supplementary Table 8: Discrepancy of MRD results when samples for NGS-MRD were sourced from blood (PI; Post Induction, PC; Post Consolidation)

Supplementary Methods

Patkar et al. Molecular MRD Detection in AML using Error Corrected NGS

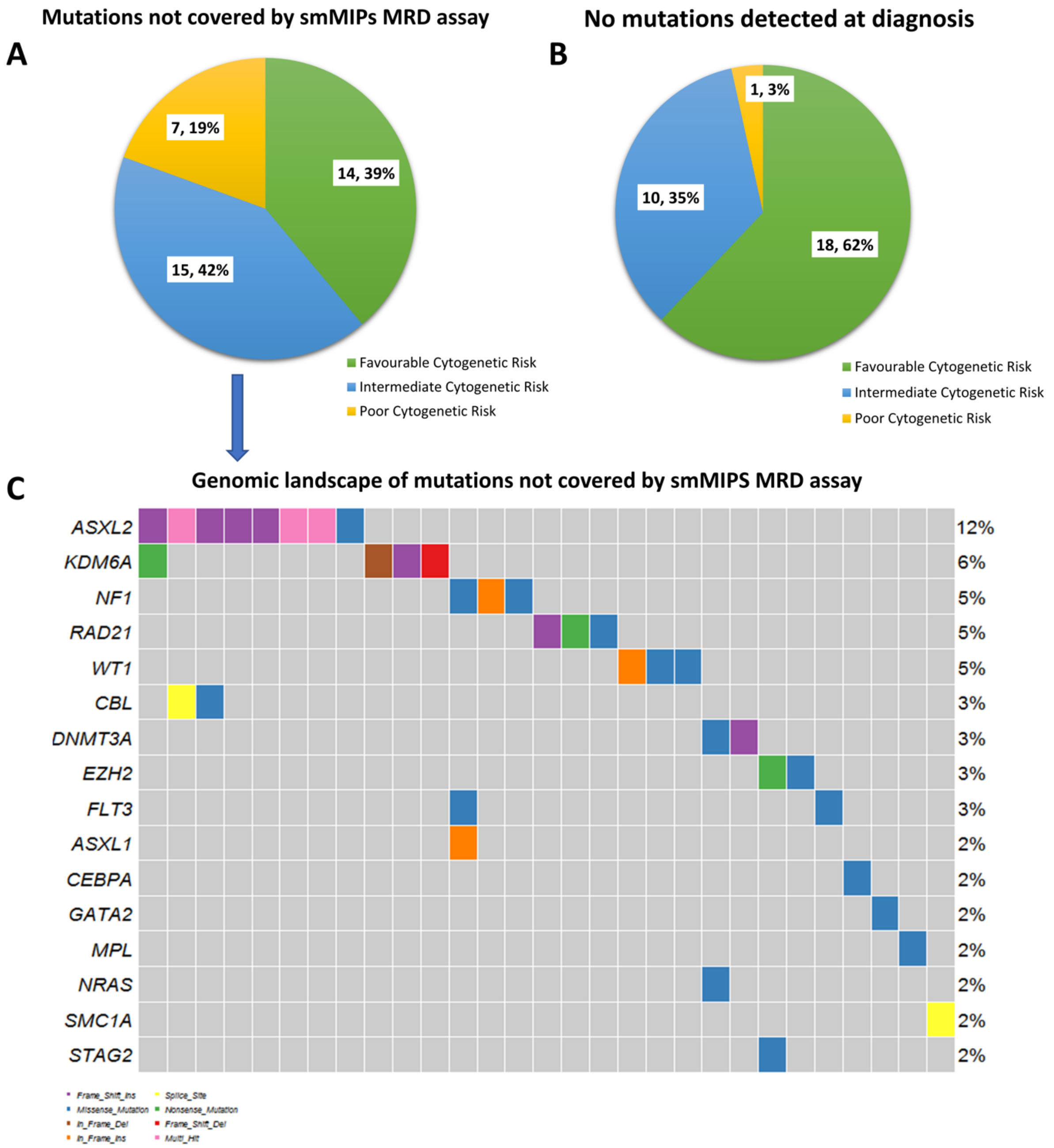

Supplementary Figure 18: Cytogenetic and genomic landscape of cases which on which NGS-MRD could not be performed. Out of 65 cases, 36 had detectable mutations (A) whereas, no mutations were detected in 29 cases (B). Genomic landscape of cases in which mutations were detected can be seen in a waterfall plot (C). Number of cases are represented in box with percentages in parenthesis. The solitary *CEBPA* mutation seen here was a case sequenced by Illumina Trusight Myeloid Sequencing Panel.

Outcome of patients in whom NGS MRD could not be performed (no mutations or not covered by panel)

A total of 65 patients (as detailed in supplementary figure 18) were excluded from NGS-MRD analysis as they lacked mutations or harboured mutations not covered by the NGS panel. Of these patients, 12 patients were refractory to chemotherapy. Of the rest (n=53), 11 patients have relapsed of which 7 have died. In addition, 10 have died of unrelated causes. The rest (n=36) are alive at last follow up.

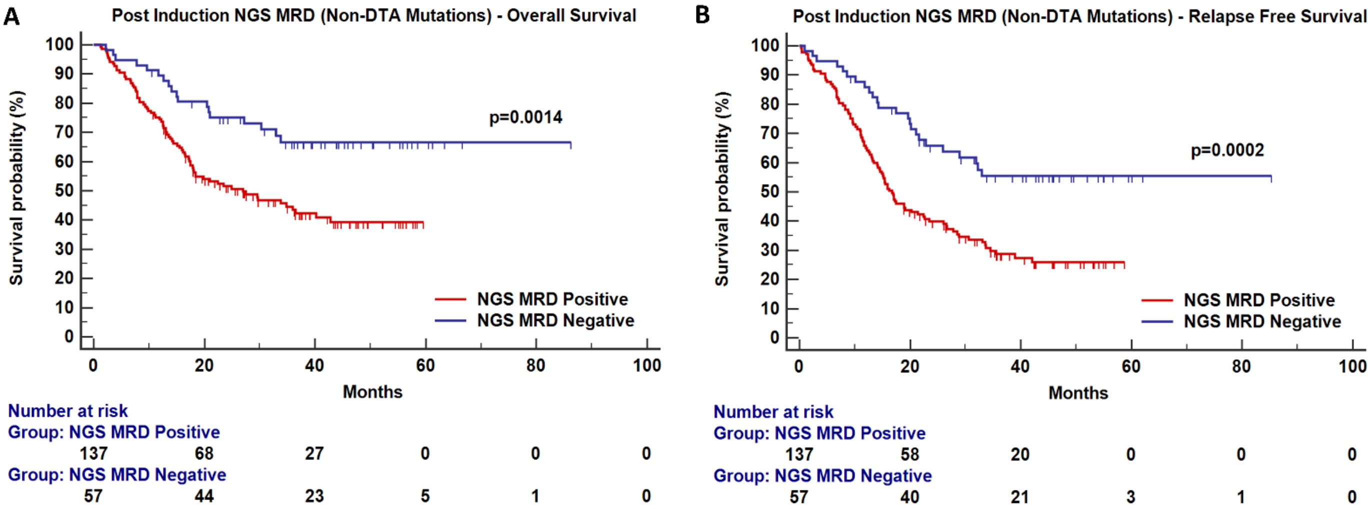

Supplementary Figure 19: Survival analysis of AML patients by NGS-MRD assessment based on non-DTA mutations after induction phases of chemotherapy. Hazard Ratio (HR) for NGS-MRD positive (non-DTA mutations): 2.25 [95% Confidence Interval (CI); 1.47 to 3.44] for OS; HR for NGS-MRD positive patients was 2.26 (95% CI; 1.56 to 3.28) for RFS.

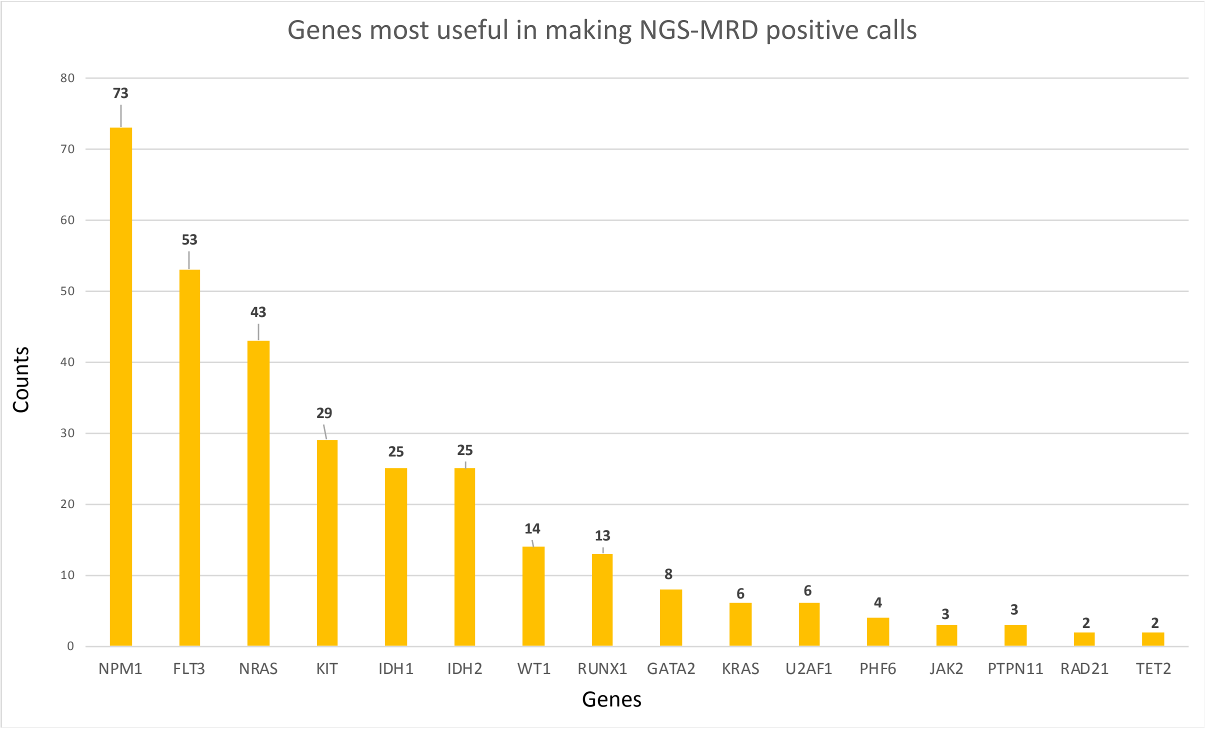

Supplementary Figure 20: This figure highlights genes that were informative in making an NGS-MRD positive call.

Supplementary References

1. Boyle EA, O'Roak BJ, Martin BK, Kumar A, Shendure J. MIPgen: optimized modeling and design of molecular inversion probes for targeted resequencing. *Bioinformatics*. 2014;30(18):2670-2672.
2. Waalkes A, Penewit K, Wood BL, Wu D, Salipante SJ. Ultrasensitive detection of acute myeloid leukemia minimal residual disease using single molecule molecular inversion probes. *Haematologica*. 2017;102(9):1549-1557.
3. Zhang J, Kobert K, Flouri T, Stamatakis A. PEAR: a fast and accurate Illumina Paired-End reAd mergeR. *Bioinformatics*. 2014;30(5):614-620.
4. Li H, Durbin R. Fast and accurate short read alignment with Burrows-Wheeler transform. *Bioinformatics*. 2009;25(14):1754-1760.
5. Li H, Handsaker B, Wysoker A, et al. The Sequence Alignment/Map format and SAMtools. *Bioinformatics*. 2009;25(16):2078-2079.
6. Wang K, Li M, Hakonarson H. ANNOVAR: functional annotation of genetic variants from high-throughput sequencing data. *Nucleic Acids Res*. 2010;38(16):e164.
7. Patkar N, Kodgule R, Kakirde C, et al. Clinical impact of measurable residual disease monitoring by ultradeep next generation sequencing in NPM1 mutated acute myeloid leukemia. *Oncotarget*. 2018;9(93):36613-36624.
8. Huang Q, Chen W, Gaal KK, Slovak ML, Stein A, Weiss LM. A rapid, one step assay for simultaneous detection of FLT3/ITD and NPM1 mutations in AML with normal cytogenetics. *Br J Haematol*. 2008;142(3):489-492.
9. Blatte TJ, Schmalbrock LK, Skambraks S, et al. getITD for FLT3-ITD-based MRD monitoring in AML. *Leukemia*. 2019;33(10):2535-2539.
